# Distribution and Toxic Effects of Antibiotics on Impacted Inland and Coastal Waters

**DOI:** 10.3390/molecules31203638

**Published:** 2026-10-12

**Authors:** Maria Garcia-Torné, Laia Hernández-Dominich, Xavier Borrell, Rachida Mazigh, Glòria Sánchez, Leena Maunula, Ankita K. Gupta, Sandra Martin-Latil, Prunelle Waldman, Marta Llorca, Marinella Farré

**Affiliations:** 1 Institute of Environmental Assessment and Water Research, C/ Jordi Girona, 18–26, 08034 Barcelona, Spain; 2 Doctoral Program in Analytical Chemistry and Environmental Science, Department of Chemical Engineering and Analytical Chemistry, University of Barcelona, 08034 Barcelona, Spain; 3 VISAFELab Laboratory, Department of Preservation and Food Safety Technologies, IATA-CSIC, 46980 Valencia, Spain; gloriasanchez@iata.csic.es; 4 Department of Food Hygiene and Environmental Health, Faculty of Veterinary Medicine, University of Helsinki, Agnes Sjöbergin katu 2, 00014 Helsinki, Finland; leena.maunula@helsinki.fi (L.M.); ankita.gupta@helsinki.fi (A.K.G.); 5 Agence Nationale de Sécurité Sanitaire de l’Alimentation, de l’Environnement et du Travail Maisons-Alfort (ANSES), 14 rue Pierre-et-Marie-Curie, 94700 Maisons-Alfort, France; sandra.martin-latil@anses.fr (S.M.-L.);

**Keywords:** antibiotic, surface inland waters, marine coastal waters, LC-HRMS, ecotoxicological risk, emerging contaminants, suspect screening, hazard quotient risk

## Abstract

Antibiotics are among the most significant medical discoveries of the 20th century. However, their extensive use and persistence in the environment have contributed to the emergence and dissemination of antibiotic resistance, causing increasing global concern. This study investigates the occurrence of antibiotics in aquatic environments to assess their ecological impact. Samples from rivers, seas and areas around wastewater treatment plants (WWTPs) in Barcelona, Valencia (Spain), Paris (France) and Helsinki (Finland), areas with different anthropogenic impacts and climate conditions, were examined to determine the occurrence of antibiotics using liquid chromatography coupled to high-resolution mass spectrometry (HPLC-HRMS). Target analysis focused on 18 compounds, alongside suspect screening for the identification of other drugs. The results were further analysed using multivariate statistical approaches, and ecotoxicological risk was assessed using the Hazard Quotient (HQ). Those most frequently detected and with the highest risk were sulfamethoxazole, metronidazole, azithromycin and tetracycline, with maximum concentration values of 69.6, 12.3, 119.1, and 12.6 µg/L, and maximum HQ values of 116,136, 6268, and 207, respectively. The highest concentrations occurred near WWTPs, while sea samples had the lowest concentrations. Overall, many compounds exceed environmental risk thresholds, demonstrating the need for improved removal treatments and stricter environmental regulation.

## 1. Introduction

Antibiotics are considered one of the greatest discoveries of the 20th century [1]. Their use has increased worldwide in both human and veterinary medicine [2], as well as playing an important role in the livestock and agriculture industries [3,4]. However, their extensive use has led to challenges, such as their impact on environmental contamination [2] and the development of antibiotic resistance (AR), due to their inadequate usage and the persistence of residues after their use. For these reasons, AR has been identified as a major global public health concern [5,6].

Antibiotics, used to treat human and veterinary infections, can reach wastewater treatment plants (WWTPs) as excreted metabolites after cleavage, glucuronidation and/or hydroxylation in bodies, although most are excreted unchanged via faeces and urine (70–90%) [7]. Once in WWTPs, antibiotics can undergo a transformation due to secondary treatment or final treatments with UV or/and chlorination. However, due to the high usage of antibiotics, the amount of this type of compound that needs to be treated in WWTPs exceeds treatment capacity. Therefore, the principal method of introducing antibiotics to aquatic media is via WWTP effluents. The continuous presence of antibiotics in the environment exerts a selective pressure that promotes the emergence and dissemination of AR among environmental bacteria. At present, the occurrence of antibiotics and antibiotic-resistant bacteria (ARB), genes (ARGs), and mobile genetic elements (MGEs) in aquatic systems is a growing global public health concern [8].

From a human health perspective, AR causes problems not only because it is an impediment to successfully treating certain infections but also due to the increased risk of mortality, alongside higher healthcare costs [9,10]. According to the European Medicines Agency (EMA), these costs can reach billions of Euros annually in Europe, while the World Health Organization (WHO) notes that AR may lead to 10 million deaths annually by 2050 if preventive and effective measures are not taken urgently [11,12,13].

Nonetheless, natural attenuation processes reduce the concentration of antibiotics and their metabolites in receiving waters through dilution, sorption to soil, sediments, and suspended solids, and chemical and biological degradation [14,15,16]. Moreover, in the WWTP, the compounds can be adsorbed into sludge, consequently releasing cleaner water but not having eliminated the contaminants [17]. For example, algae and their extracellular organic matter can act as photosensitizers by promoting indirect photolysis and degradation of antibiotic residues [18]. This natural process can effectively eliminate antibiotics from water bodies, minimising their adverse impact on aquatic organisms and reducing the spread of AR [19]. This biodegradation/biotransformation process, which may occur not only in WWTPs but also in natural aquatic environments, can generate toxic by-products and make it difficult to evaluate the fate of the initial antibiotic. Antibiotic residues and their known transformation products (TPs) have been found in surface inland waters around the world, ranging between low pg/L and ng/L and in coastal marine waters up to µg/L [20]. Furthermore, seasonal fluctuations in antibiotic residues are important, as concentrations are expected to be higher in winter than in summer [21]. This is expected because antibiotic consumption varies by season, rising in winter, and because degradation processes slow in winter (fewer hours of light reduce photodegradation, and lower temperatures slow microbial activity and biodegradation) [22,23].

Antibiotics and their TPs affect the aquatic environment by exerting a selective pressure that promotes the emergence and dissemination of antibiotic resistance genes (ARGs) among free-living bacteria (which cause delayed nitrite oxidation, metagenesis, and increased toxicity of various chemical combinations [24,25]). However, their continuous discharge also introduces other problems such as bioaccumulation in organisms from receiving waters and ecotoxicological effects by altering functions and metabolism in invertebrates or fish [14,26,27]. For example, antibiotics accumulated in fish can cause oxidative stress, histopathological lesions, reproductive disorders, and genotoxicity at low concentrations, among others [8,28]. Lower trophic levels like invertebrates exhibit low acute toxicity, but chronic exposure induces more toxic effects [29,30,31]. In algae and aquatic plants, antibiotics inhibit photosynthesis and increase oxidative stress, and macrolides and sulphonamides can also adversely affect algal development [8,32].

Without mandatory monitoring requirements, antibiotic residues released from various sources (WWTPs, pharmaceutical manufacturing, agriculture) into the environment may go unchecked, perpetuating antibiotic pollution in water, soil, and other environmental compartments [8,33]. To address these challenges, researchers have developed several monitoring and prioritisation frameworks. For instance, the WHO AWaRe classification promotes appropriate antibiotic use and monitoring to support antimicrobial stewardship. At the same time, the European Watch List identifies substances, including antibiotics, that require environmental monitoring because of their potential ecological impact and contribution to AR [34,35]. Recently, the European Union (EU) included two more antibiotics in the latest Watch List revision [36], including the tetracyclines oxytetracycline (widely used in aquaculture) and tetracycline (included in the Stockholm list of environmentally harmful Active Pharmaceutical Ingredients (APIs)), while emphasising the importance of also monitoring macrolides (roxithromycin, tylosin), sulphonamides (sulfamethazine, sulfadiazine) and quinolones (norfloxacin) based on their possible contribution to AR. Furthermore, Directive 805/2026 recently included three antibiotics (azithromycin, erythromycin, and clarithromycin) in the priority substance list; therefore, these macrolides will be periodically monitored in surface waters at the European scale, and their levels will help determine the good or bad chemical status of surface water bodies.

Based on previous research [21], the main objectives of this study were to investigate the occurrence of a wide range of antibiotics (including macrolides, fluoroquinolones, lincosamides, nitroimidazoles, sulphonamides, tetracyclines and diamino pyridines) in impacted inland surface waters and marine coastal waters coming from different weathering and human pressures using a suspect screening approach and to evaluate whether the concentration levels found pose a potential risk to the environment. For this reason, samples from southern Europe (Barcelona and Valencia, Spain), central Europe (Paris, France) and northern Europe (Helsinki, Finland) were collected to distinguish concentration patterns by the different pressures they receive.

## 2. Results and Discussion

### 2.1. Analytical Results

#### 2.1.1. Suspect Screening

**Figure 1:**
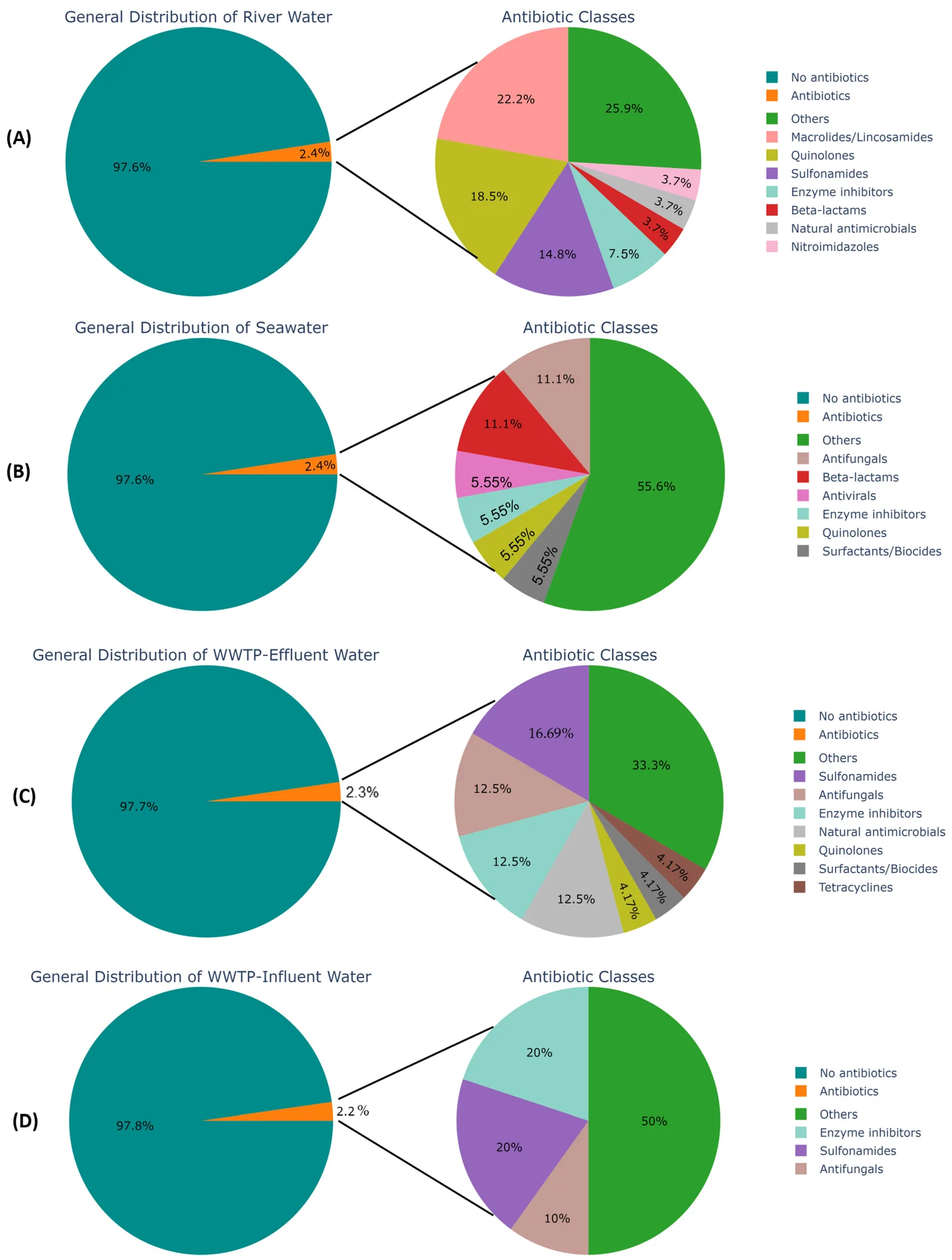
Distribution of tentatively identified antibiotics and their classes in (**A**) river water samples, (**B**) seawater samples, (**C**) WWTP effluent, and (**D**) WWTP influent.

After processing the data, 2.4% of the tentatively identified compounds in river samples were antibiotics. Among them, the most relevant groups were macrolides, quinolones, and sulphonamides, such as lincomycin, clarithromycin, ciprofloxacin, norfloxacin, sulfamethaxine, and sulfamethoxazole, all of which were present in French samples (Figure 1A). In seawater samples, the same percentage represented antibiotics (2.4%); however, the classification and distribution differed slightly. Other compounds, such as natural compounds with antibiotic properties, intermediates, and preservatives used in antibiotic synthesis, were highly represented, followed by other antibiotics like β-lactams (nafcillin) in Barcelona (Figure 1B). 

In WWTP effluent (Figure 1C) and influent (Figure 1D), around 2.2% of the tentatively identified compounds were classified as antibiotics. Moreover, a high percentage of the suspects in influent and effluent samples remained unclassified, being about 33% and 50%, respectively. In French and Finnish samples, influent and effluent showed sulphonamides (sulfamethoxazole, sulphapyridine, sulfamethazine, among others). Remarkably, the effluent contained more compound diversity than the influent, but sampling did not account for retention time; the influent and effluent were not paired. During treatment, each compound could have formed different transformation products. So, in this graph, we compare relative percentages rather than absolute values. However, if an effluent showed a higher presence of a compound than its paired influent, this relates to chemical diversity rather than signal intensity.

#### 2.1.2. Target Analysis

Table S1 shows the detection frequency for each compound based on the total number of samples, and Table S2 shows the concentrations found. The results show that in river samples, antibiotics were present at the µg/L level, although they were detected in most analysed samples. This phenomenon may be due to human activity and discharge from close or upstream WWTPs. In contrast, in the Barcelona Sea samples, only two antibiotics were detected, which may be explained by greater dilution in the marine environment.

**Figure 2:**
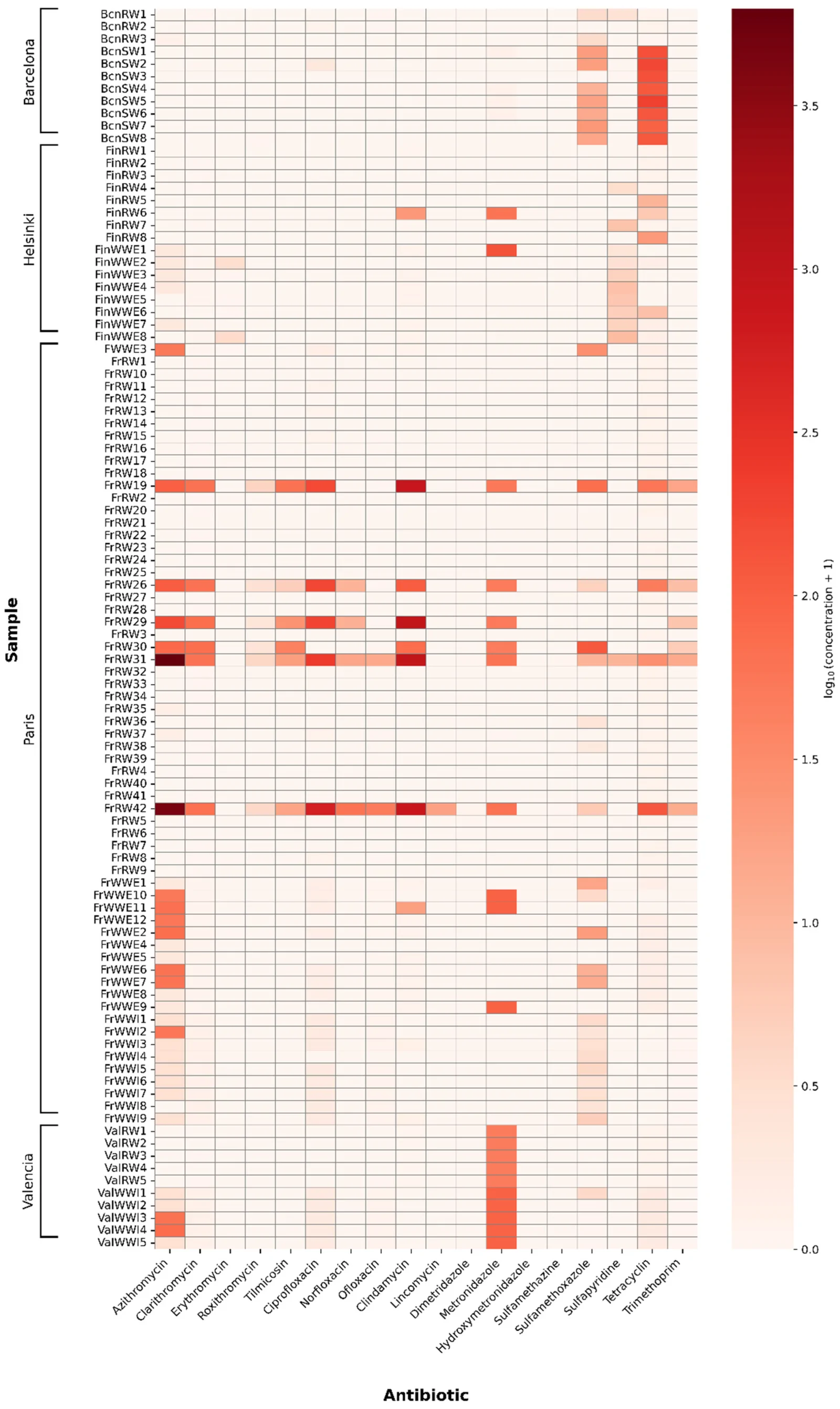
Distribution of antibiotic concentrations detected in water samples, represented in logarithmic scale.

Among the antibiotics quantified, the most frequently detected compounds were sulfamethoxazole (32%), metronidazole (21%), and to a lesser extent, azithromycin and tetracycline (16%). Moreover, the highest concentrations were detected for azithromycin (119.1 µg/L), sulfamethoxazole (69.65 µg/L), and tilmicosin (62.24 µg/L). These extreme levels were observed in influent samples and attenuated during wastewater treatment and environmental dilution. Figure 2 shows the quantification of the compounds identified at a confidence level of 1 in the form of a heatmap. Furthermore, Table S3 in the Supplementary Materials shows the limits of detection and quantification.

Comparing the different sample matrices and locations, the samples from the Seine River (France), especially “FrRW42” (Located down the Seine) and the ones located at “SeineAmont”, are the ones with the highest variability of antibiotics and the highest concentrations. The “FrRW19” (downstream of the Marne) sample also shows high antibiotic diversity, with especially high concentrations of tilmicosin (62.24 µg/L) and sulfamethoxazole (41.39 µg/L). In Barcelona’s samples, although made up of still surface water, concentrations were much lower, as mentioned before, due to dilution. Moreover, for some detected antibiotics, the detection frequency between the WWTPs’ influent and effluent was sometimes reduced. For instance, for azithromycin, the detection frequency in influent samples (11%) was lower than in effluent (53%). These observations could be explained by the degradation of azithromycin–glucuronide during WWTP treatment, after which the isolated antibiotic was detected in the effluent. However, it could also be due to differences in the water portion sampled, which was not the same because of hydraulic retention time [37].

Figure S1 shows the detected concentrations across all matrices. In seawater samples (Figure S1A), tetracyclines, sulfamethoxazole, and metronidazole were the most frequently observed antibiotics. In contrast, river water samples from Paris had the highest concentration peaks, while those of other locations had lower quantities and less diversity (Figure S1B). Metronidazole should be noted in the Valencian samples, as it occurred at significant concentrations only in this area. Finally, near WWTPs, azithromycin, metronidazole, and, to a lesser extent, sulfamethoxazole and sulphapyridine were notably present (Figure S1C).

The highest concentrations were in azithromycin (119.10 µg/L), sulfamethoxazole (69.65 µg/L), tilmicosin (62.24 µg/L), clindamycin (40.40 µg/L), ciprofloxacin (34.28 µg/L), and lincomycin (33.46 µg/L), all from river surface water in France. The first three show log(Kow) values between 0.0 and 4, indicating moderate to high hydrophobicity, with a tendency to partition into organic phases, while still being detectable in water. However, because log(Kow) does not account for pH-dependent ionisation, logD values at pH 7.4 were considered environmentally relevant. Values can differ considerably from log(Kow), potentially overestimating the tendency of some ionizable compounds to partition into organic phases under the considered pH conditions. Table S4 summarises experimental and predicted values. The logD (pH 7.4) values for azithromycin, sulfamethazine, and tilmicosin were 2.7, −0.5, and 0.8, respectively. These values represent markedly different partitioning behaviour in environmental water samples. In contrast, other compounds like lincomycin, which had a low log(Kow) of 0.2, were more likely to be found in water than in sediments and were less persistent, with a short half-life (below 1 day) [38].

The tendency of some compounds to migrate to organic matter highlights the relevance of considering the WWTPs’ sludge as a potential reservoir of contaminants. Thus, the stability of some compounds in organic matter and sediments, rather than in water, should not be considered as complete removal from the environment. Moreover, sewage sludge may be used in agriculture as a fertiliser, in composting, or incinerated or disposed of in landfills and may then be reintroduced into the environment [39].

Across all compounds, macrolides like azithromycin, clarithromycin, and tilmicosin showed significant concentrations in several locations. Fluoroquinolones like ciprofloxacin and norfloxacin were less ubiquitous, but high concentrations were reported in certain specific points. Nitroimidazoles, especially metronidazole, were highly present in samples from Valencia and, in small proportions, in specific samples from Paris and Helsinki. Sulfamethoxazole was quantified across a wide distribution, especially in samples from the Seine River (France) and some from the Marne River (France). Finally, tetracyclines and diaminopyrimidines occurred sporadically in river samples, with the former standing out for their significant amounts in seawater. A literature review reported concentrations reaching µg/L in urban surface waters and, especially, in effluent water from WWTPs [40,41]. In samples from France and Valencia, some antibiotics were found at high concentrations. Moreover, seawater samples, or those farther from WWTP discharge points, showed much lower levels (<1 µg/L) [42].

Other parameters that might be important for comparing the results include regional differences, which are also linked to antibiotic consumption patterns, population density, the type of WWTP and the treatments used, and the sampling season. Moreover, the sampling strategy, even if similar, can differ slightly between sites. According to the European Centre for Disease Prevention and Control (ECDC), the total consumption of antibiotics in 2024 shows France in a 2nd position, Spain in the 5th, and Finland in the 23rd position, with 26.5, 24.2, and 13.7 DDD (Defined Daily Dose) per 1000 inhabitants per day, respectively [43]. However, the potential availability of antibiotics without a prescription should also be considered. In all three countries, antibiotic drugs are considered prescription-only medicines. However, studies confirm that non-prescribed (over-the-counter) sales still occur [44,45]. Moreover, France recently implemented specific protocols allowing pharmacists to dispense certain antibiotics under well-defined conditions following pharmacy-based diagnostic testing, without a prior medical prescription [46].

Moreover, other parameters, such as meteorological conditions, further influence the results. In this case, Barcelona’s sampling followed two weeks of continuous rainfall, likely reducing the detected concentrations, whereas French samples were collected during a drier period. In Finland, higher rainfall and dilution generally resulted in lower concentrations. 

Among the studied compounds, some have already been a focus and are included in different lists to monitor their presence and use. Azithromycin, clarithromycin, erythromycin, ciprofloxacin, clindamycin, metronidazole, and trimethoprim are listed in the WHO Model List of Essential Medicines 2025, which classifies them into Access, Watch, or Reserve medicines (AWaRe watch list). Among them, the first four are in the Watch category, while the others remain in Access [47].

Overall, the findings suggest that WWTPs are possible major entry points for antibiotics into the environment, as these compounds are not completely removed during wastewater treatment. The high antibiotic concentrations in the influent are mainly attributable to hospital discharges. However, as expected, concentrations remain much lower in surface waters due to dilution. Nevertheless, complementary analyses of antibiotic TPs as well as ARGs, which may be more persistent than the antibiotics themselves, are needed to assess the environmental risk of AR fully.

### 2.2. Statistical Analysis

**Figure 3:**
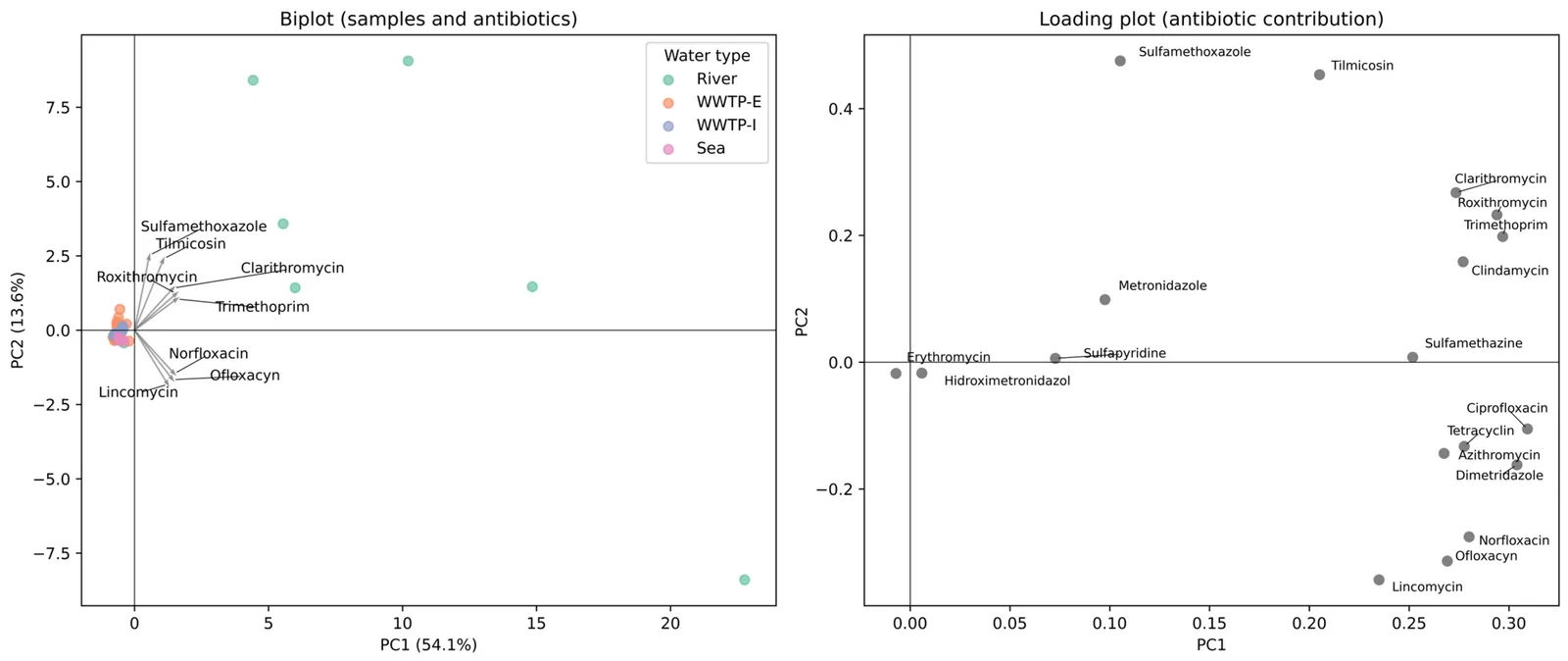
PCA of water samples, score plot (**left**), and loading plot (**right**).

To assess possible patterns among the samples, we performed a Principal Component Analysis (PCA) (Figure 3). The first two components explained about 70% of the variability, with river samples clearly separated from the others, reflecting distinct concentration profiles. Sulfamethoxazole, clarithromycin, tilmicosin, roxithromycin, and trimethoprim were the main contributors to this separation, while this principal component had little influence on compounds such as erythromycin and metronidazole.

**Figure 4:**
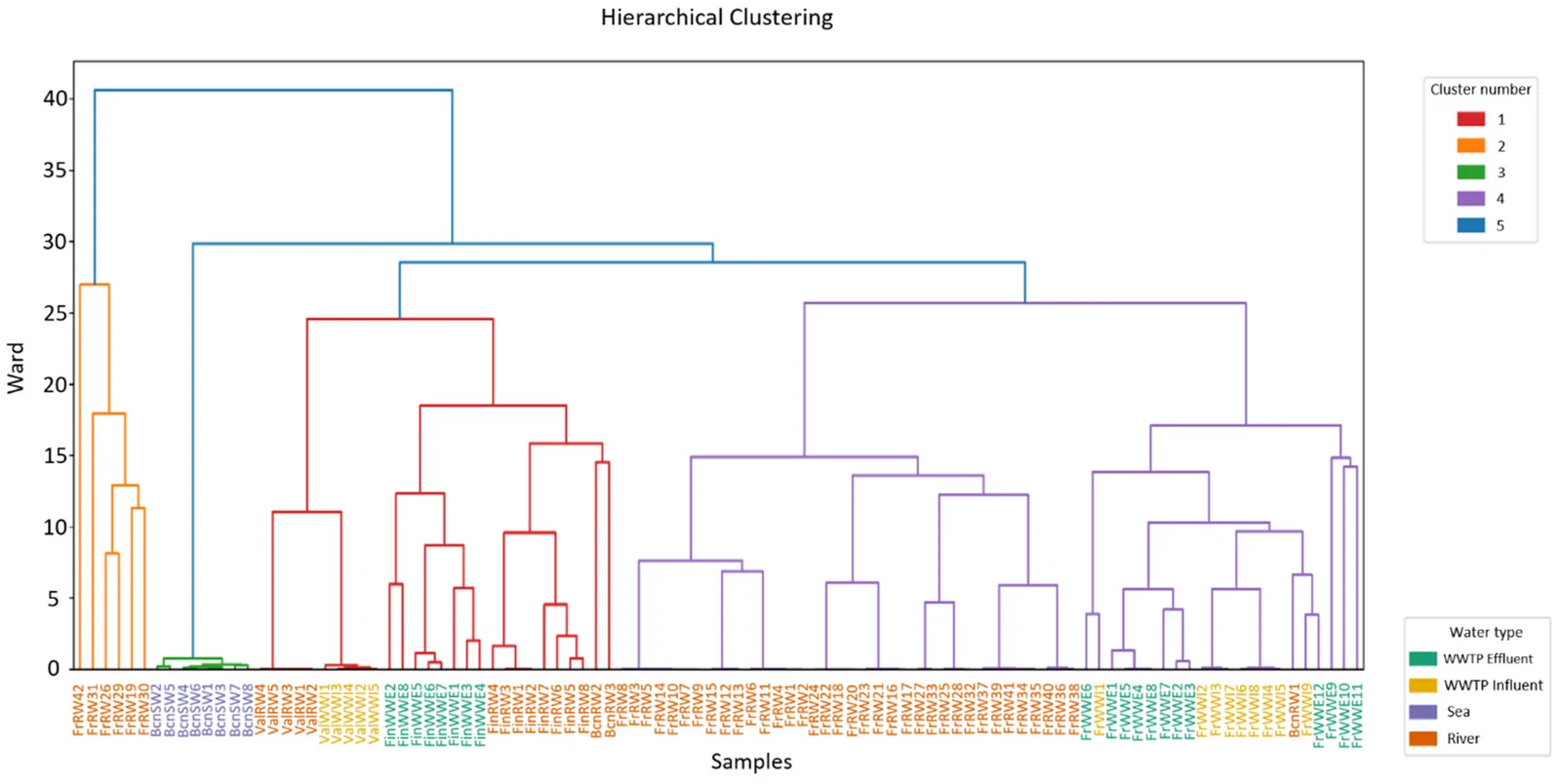
Hierarchical clustering using Ward’s method of classifying water samples, coloured by water type and clustering.

Figure 4 shows a hierarchical cluster analysis using Ward’s method applied to antibiotic concentrations and environmental factors. It revealed distinct groupings by water matrix and concentration profiles, as well as by environmental conditions across locations. The orange cluster grouped Parisian samples (“SeineAval”, “SeineAmont”, “MarneAval”) characterised by particularly high antibiotic levels. Seawater samples formed a separate green cluster, reflecting the low concentrations. A red cluster combined samples from Valencia and Finland, including both river water and WWTP effluents, together with two Barcelona River samples showing similar characteristics. The purple cluster grouped the remaining Paris River samples with their influent and effluent counterparts, along with one additional Barcelona River sample.

Finally, to evaluate which factors had the greatest influence, we performed an ANOVA. Population-level analysis produced no significant results because of the limited sample diversity. So, we applied the test by water matrix (seawater, river water, WWTP influent/effluent) (Figure S2). The comparative boxplots show that erythromycin, metronidazole, and sulphapyridine differed significantly (*p* < 0.05), with higher concentrations typically found in wastewater. In contrast, seawater samples consistently exhibited the lowest values, reflecting strong environmental dilution, while river samples showed variable distributions depending on the compound. Together with the PCA and clustering results, these findings provide a solid basis for interpreting the sources and persistence of antibiotics in aquatic environments.

### 2.3. Environmental Risk Assessment

To assess whether the detected antibiotics could pose an environmental threat, we estimated their ecotoxicological risk using HQ values. The HQ evaluates ecological toxicity, not AR, although the information it provides can help identify the most toxic compounds for the environment. Tables S5 and S6 in the Supplementary Materials summarise the data used.

The HQ values (Table S5) show that the compounds found in seawater, tetracycline, and sulfamethoxazole represent a high ecotoxicological risk (HQ values up to 207.40 and 22.18, respectively) (Table S6). In rivers, samples can be differentiated into different groups. However, all samples had high HQ values for tetracycline (up to 125.66). In Valencian surface waters, metronidazole also had a high impact and posed high risk in all the samples (HQ values up to 48.06), even higher than tetracycline (0.21). In other surface water samples from Barcelona and Helsinki, we detected other compounds at high-risk values (azithromycin, ciprofloxacin, clindamycin, metronidazole, sulfamethoxazole, and sulphapyridine). 

**Figure 5:**
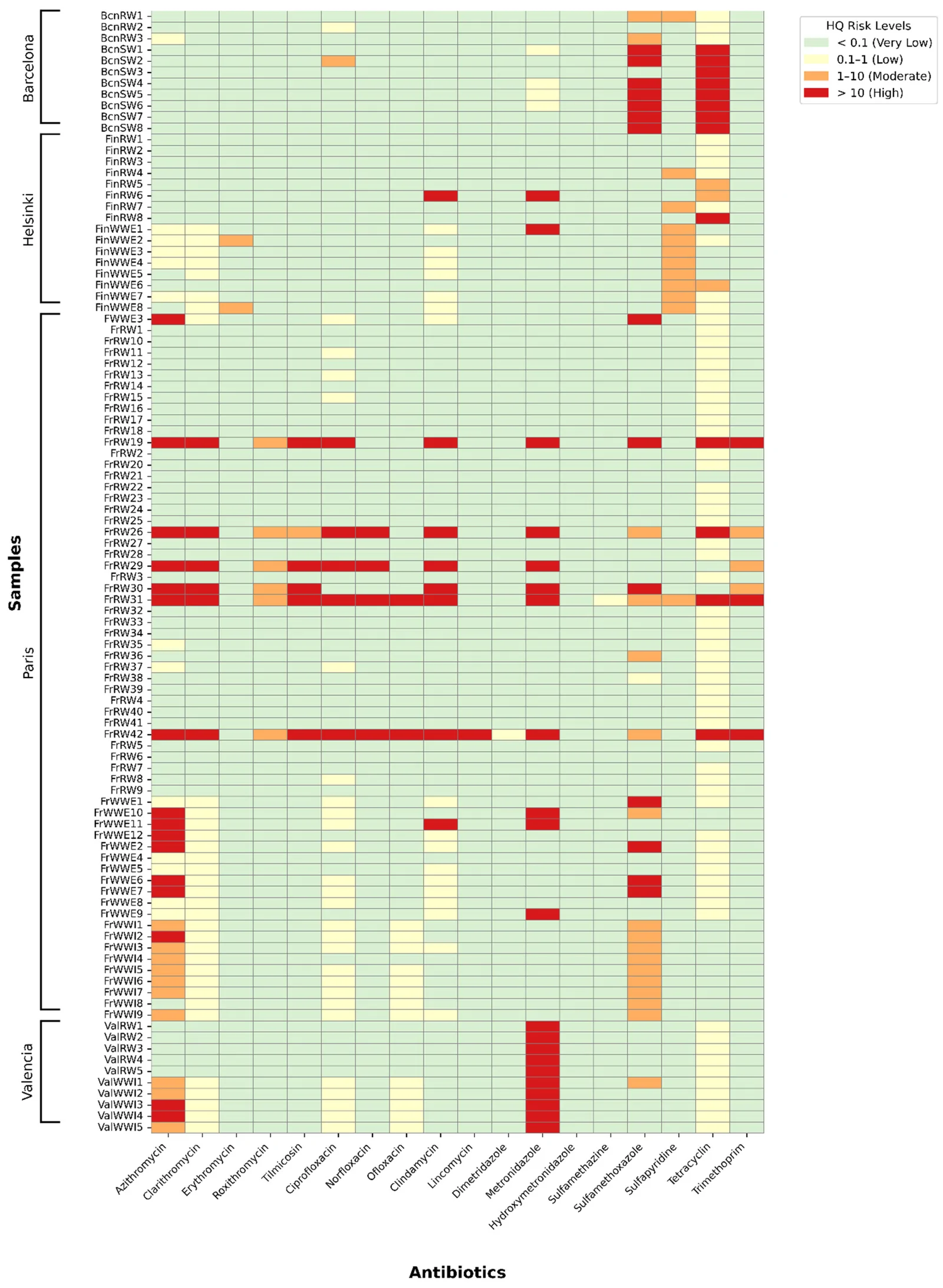
Heatmap of HQ values.

Regarding the French surface water samples, there were some specific samples with very high values: FrRW19 (presented a high risk for 10 of the compounds), FrRW26 (11 compounds), FrRW29 (9 compounds), FrRW30 (8 compounds), FrRW31 (14 compounds), and FrRW42 (14 compounds). Near WWTPs, the effluent samples presented a higher risk than influent samples. Azithromycin, metronidazole, and sulfamethoxazole had the highest values. However, azithromycin, clarithromycin, ciprofloxacin, ofloxacin, sulphapyridine, and tetracycline showed high values in all samples. The heatmap in Figure 5 shows the critical contamination points and the compounds with higher risk, with trends and patterns depending on sample type.

Ecotoxicological impacts differ by antibiotic class. For example, macrolides inhibit bacterial growth and affect aquatic organisms; fluoroquinolones bioaccumulate and reduce WWTP efficiency; lincosamides alter microbial composition; nitroimidazoles show genotoxicity; sulphonamides disrupt nitrogen processes; tetracyclines are toxic to primary producers and invertebrates; and trimethoprim enhances sulphonamide toxicity. Overall, these results stress the importance of risk assessment in human-impacted waters and WWTP effluents to identify critical contamination hotspots and prioritise mitigation strategies.

## 3. Materials and Methods

### 3.1. Chemicals and Reagents

We selected the compounds according to our previous study [21]. The high-purity standard compounds and the isotopically labelled compounds, used for confirmation and quantification purposes, were supplied by Sigma-Aldrich (St. Louis, MO, USA). Further information about the compounds can be found in Supplementary Materials Table S7. The mix of antibiotics was prepared from individual 1000 mg/L stock solutions, prepared previously in the optimum solvent for each compound, to have a final concentration of 5 mg/L. Similarly, the mix of isotopically labelled compounds was prepared for internal standard monitoring and normalisation during sample extraction, preconcentration and analytical steps. When possible, quantification was based on an external calibration curve prepared in methanol–HPLC water (1:1). All the solutions were kept at −20 °C to ensure their stability and prevent degradation. 

HPLC-grade purity solvents such as methanol, water, and acetonitrile were obtained from LiChrosolv^@^ Merck (Darmstadt, Germany). We purchased hydrochloric acid for sample pretreatment from PanReac AppliChem (Murcia, Spain) and EDTA, from Sigma-Aldrich (St. Louis, MO, USA), both with a purity above 95%.

### 3.2. Case Studies

To monitor antibiotics and their distribution patterns shaped by weathering and human pressures, we selected sampling sites across Southern, Central and Northern Europe. In the first case, samples were taken from a site highly impacted by human activity, i.e. the Barcelona metropolitan area (Spain), and included inland surface waters from the surrounding rivers (the Llobregat and Besós rivers) and seawater from several urban beaches (Forum Beach, Levante Beach, Nova Mar Bella Beach, Mar Bella Beach, Bogatell Beach, Nova Icària Beach, Somorrostro Beach, and Sant Miquel Beach). These beaches were considered potential final sinks for river discharges carrying antibiotics and ARGs, if present in the surrounding rivers. Additionally, we collected samples from the protected natural area of L’Albufera of València (Spain) to evaluate contaminant transfer from WWTPs discharges into the lagoon, including WWTP influent and effluent samples, as well as surface water from L’Albufera.

**Figure 6:**
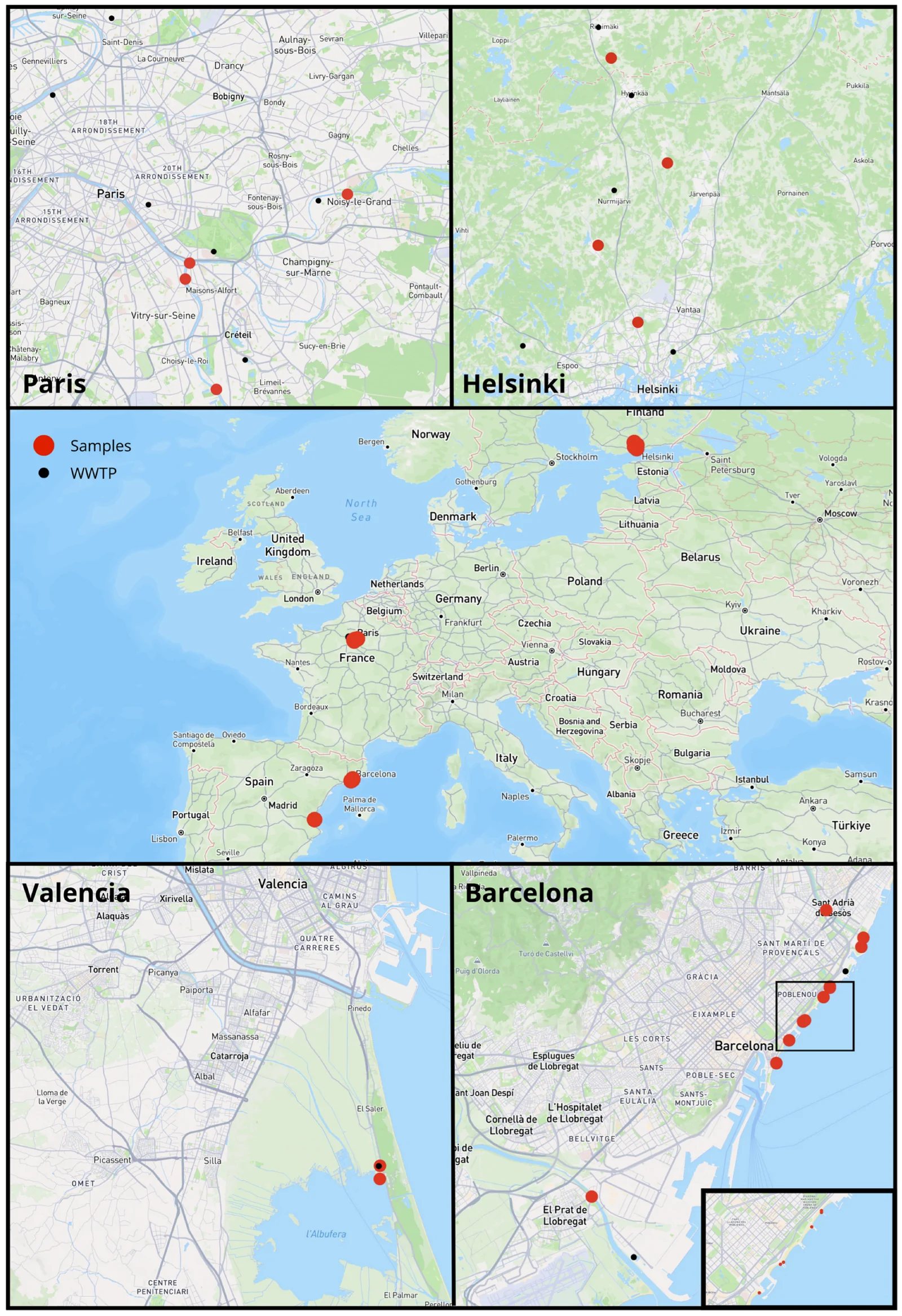
Study area of the samples collected in Barcelona, Valencia (Spain), Helsinki (Finland), and Paris (France) represented by red dots. The surrounding WWTPs in the study area are represented in black.

For Central Europe, we collected influent and effluent samples from WWTPs, as well as surface river water samples from the Paris (France) metropolitan area along the Seine River and Marne rivers. Finally, as an example from Northern Europe, we also collected WWTP effluent samples, along with surface water samples from the River Vantaa and its tributaries, (Finland). Of the 100 samples collected, 66 were river samples, 17 effluent WWTP samples, 9 influent WWTP samples, and 8 sea samples. Figure 6 shows their locations. 

Sampling (March 2024–2025) was carried out using glass bottles previously rinsed with ultrapure water, methanol, and acetone. At each sampling site, we rinsed the bottles with the corresponding water sample before filling them, leaving some headspace to prevent breakage during freezing. We then shipped the samples under refrigeration to IDAEA-CSIC for extraction and analysis. Sample volumes depended on the water origin: 200 mL for seawater, 100 mL for surface river water and effluent WWTP water, and 25 mL for influent WWTP water. The samples were preserved by freezing until analysis within the first week after sampling.

The samples were encoded as follows: Bcn, Val, Fr, and Fin correspond to Barcelona, Valencia, France, and Finland, respectively. The water types WWE, WWI, SW, and RW, correspond to wastewater effluent, wastewater influent, seawater, and river water, respectively. Finally, samples of the same type are numbered.

### 3.3. Sample Pretreatment

Sample preparation was based on the methodology described in the literature [48], with minor modifications. First, 10 µL of an internal standard solution was added to correct possible matrix effects and instrumental errors. Next, samples were filtered through 0.7 µm filters, and hydrochloric acid (45%) was added drop by drop to achieve acidic conditions (pH 2.5). Afterwards, a 0.1 M of EDTA (in water) solution was incorporated to reach a final concentration of 0.1 % to minimise macrolides’ chelation. Then, the samples were agitated vigorously in a vortex, and solid phase extraction (SPE) was carried out using Oasis HLB cartridges (200 mg, 6 mL) at a flow rate of c.a. 1 mL/min in a manifold. The procedure for SPE first included conditioning with 2 × 2 mL of methanol, followed by 2 × 2 mL of HPLC water, both under gravity conditions. Subsequently, the samples were loaded using a vacuum system. Then, the cartridges were further dried under a vacuum system for 30 min. Immediately thereafter, the cartridges were eluted using 2 × 2 mL of methanol under gravity conditions. The obtained extracts were evaporated under a gentle nitrogen stream to near 1 ml, and then they were transferred to HPLC vials and further evaporated to dryness. Finally, the extracts were reconstituted in 1 mL of a methanol–HPLC water (1:1) solution, vortexed, and kept at −20 °C until their instrumental analysis.

### 3.4. Instrumental Analysis

The samples were analysed using high-performance liquid chromatography coupled to high-resolution mass spectrometry (HPLC-HRMS). The equipment consisted of a liquid chromatography Aquity LC system (Waters^®^, Milford, MA, USA) which was equipped with a C_18_ analytical column (Hibar^®^ HR 50-21 Purospher^®^ STAR RP-18 end-capped column, Merck KGaA, Darmstadt, Germany) (3 μm, 2 mm × 125 mm). The chromatographic separation was performed under gradient conditions using (A) acetonitrile and (B) HPLC water with 0.1 % formic acid as mobile phases.

The gradient started at 10 % A, increased to 99 % in 8 min, was maintained for 10 min, and returned to initial conditions at the end of 12 min. Finally, the conditions were maintained for one more minute. The flow rate used was 0.2 mL/min. The chromatographic system was coupled to a Q-Exactive mass spectrometer (Thermo Fisher Scientific, San Jose, CA, USA) equipped with an electrospray ionisation source (ESI) operating in positive mode. Data were acquired in full scan mode at a resolution of 70,000 full width at half maximum (FWHM) from 100 to 1000 *m*/*z* and, in parallel, by data-dependent scanning for the 10 most intense ions at a resolution of 30,000 FWHM. Xcalibur 4.0 software controlled the entire system. Table 1 summarises all the experimental conditions.

### 3.5. Data Processing

We processed the data using Compound Discoverer software version 3.3 SP1 from Thermo Fisher Scientific to detect antibiotics and related compounds such as TPs. We compared the initial tentative compounds with two online databases: ChemSpider for structural information and MzCloud for the mass spectral data. In addition, we added the *Antibiotics_ITN_MSCA_ANSWER_160616_WDTXSIDS* list from the NORMAN database to detect compounds identified as antibiotics and their main TPs. We filtered the results according to Schymanski’s confidence levels [49]. Figure S3 shows the scheme of the levels.

Finally, for those compounds tentatively identified at a confidence level of 2 for which a pure standard was available in the laboratory, the data were processed with Xcalibur Quan Browser version 4.1 (Thermo Fisher Scientific, San Jose, CA, USA) for target analysis and final identification at a confidence level of 1.

### 3.6. Antibiotics Risk Assessment

The bioaccumulation of antibiotics and their potential effects on the environment can be estimated using the Hazard Quotient (HQ), which allows the evaluation of the potential ecological risk associated with their presence in the aquatic environment [14,50]. The HQ is calculated as the ratio between the measured environmental concentration of the studied antibiotic (Ca) and its respective Predicted No Effect Concentration (PNEC), obtained from the NORMAN Ecotoxicology Database [51].

(1) $$\mathrm{HQ}=\frac{\mathrm{Ca}}{\mathrm{PNEC}}$$

If the HQ value is below 0.1, no adverse effect is expected. If the values are between 0.1 and 1, the risk is low. If the values are between 1 and 10, the risk is moderate. HQs greater than 10 indicate high risk, and a significant possibility of adverse effects is predicted [52]. Accordingly, we estimated the HQ for each compound at each sampling point to determine whether its presence may represent a potential ecological risk for aquatic organisms.

## 4. Conclusions

In this study, suspect screening was carried out to evaluate the presence of antibiotics in 100 inland waters from different European countries, covering a range of different climates, and coastal waters, with a subsequent target analysis; the results showed that the most frequently detected ones were sulfamethoxazole, metronidazole, azithromycin, and tetracycline. Concentrations varied significantly among samples, as expected, potentially reflecting different regional consumption and/or prescription patterns as well as different meteorological and hydrological conditions. In sea samples, values are lower than in the others due to dilution. However, concentrations and ecological risk are higher in samples from rivers and near WWTPs.

Statistical analyses revealed that the samples can be grouped by antibiotic profile or water type, providing important information for future studies. This allows for the grouping of different sample types and extrapolation of identified patterns to similar conditions and locations.

HQs were calculated to support the analysis, and in several samples, a significant ecological risk was found for compounds such as metronidazole, sulfamethoxazole, tetracycline, and azithromycin, often exceeding the HQ > 1 threshold.

The conclusions from this research show that wastewater treatment technologies are not sufficient to remove the complex mixture of antibiotic drugs and the TPs they receive. Therefore, it is necessary to improve both water treatment and the disposal of these compounds. Therefore, continuous monitoring and stronger regulations are important if we are to reduce their environmental impact and the possible spread of AR. Over the coming years, quaternary treatments, which offer enhanced removal efficiency for organic micropollutants, are expected to be implemented across the European Union in systems serving >150,000 population equivalent (p.e.), following the enactment of Directive (EU) 2024/3019. Such treatments will likely contribute to the reduction of antibiotic levels described in the present study. Nevertheless, when implementing such quaternary treatments in smaller agglomerations (10,000–150,000 p.e.), ecological risk assessment exercises should guide prioritisation and account for several antibiotic families as relevant indicators, given the significant HQs described in the present study.

## Figures and Tables

**Table 1:** Operational conditions for the antibiotic analysis.

	Condition	Value
1	Chromatographic column	C18 analytical column (Hibar^®^ HR 50-21 Purospher^®^ STAR RP-18 end-capped column) (3 μm, 2 mm × 125 mm)
2	Mobile phase	A: Acetonitrile; B: H_2_O + Formic acid 0.1%
3	Flow rate	0.2 mL/min
4	Sample temperature	15 °C
5	Detection mode	Full Scan
6	Injection volume	10 µL
7	Ionization source	ESI Positive
8	Gradient	10–99%
9	Mass range	100.0–1000.0 *m*/*z*

## Data Availability

The original contributions presented in this study are included in the article/Supplementary Materials. Other data will be available through the ONE-BLUE CECs-DB. Further inquiries can be directed to the corresponding authors.

## Supplementary Materials

The following supporting information can be downloaded at: https://www.mdpi.com/article/10.3390/molecules31203638/s1, Figure S1. Antibiotic concentrations detected in sea (A), river (B), and near WWTPs (C) water samples. Figure S2. Boxplots showing antibiotic concentrations across different water types with ANOVA p-values (*p*-values in red* are statistically significant). Figure S3. Confidence levels for the Suspect screening identification. Table S1. Detection frequency and concentration levels of antibiotic drugs in rivers, sea and samples collected near WWTPs. Table S2. Detailed concentrations (µg/L) of the analysed antibiotics. Table S3. Calculated limits of detection (LOD) and quantification (LOQ) for each antibiotic in each water matrix. Table S4. Summary of predicted no-effect concentrations (PNECs), octanol-water partition coefficients for the selected compounds. Table S5. Detailed Hazard Quotient (HQ) of the analysed antibiotic in each water sample, with colour coding indicating risk levels (green, yellow, orange, red—from lowest to highest). Table S6. Detection and risk frequency of antibiotics analysed by target analysis in rivers, sea and samples collected near WWTPs. Table S7. Selected antibiotics and their internal standards parameters.
