# Supplementary material for: Distribution and Toxic Effects of Antibiotics on Impacted Inland and Coastal Waters
Source: Molecules. 2026 Oct 12;31(20):3638. doi: 10.3390/molecules31203638 (PMC13707889; doi:10.3390/molecules31203638)
Supplement: Supplementary file 1 [file molecules-31-03638-s001.zip › molecules-4556375-supplementary.pdf]

SUPPLEMENTARY MATERIAL

DISTRIBUTION AND TOXIC EFFECTS OF ANTIBIOTICS ON IMPACTED INLAND AND COASTAL WATERS

Maria Garcia-Torné[1,2], Laia Hernández-Dominich[1], Xavier Borrell[1], Rachida Mazigh[1], Glòria Sánchez[3], Leena Maunula[4], Ankita K. Gupta[4], Sandra Martin-Latil[5], Prunelle Waldman[5], Marta Llorca[1,*] & Marinella Farré[1,*]

[1]Institute of Environmental Assessment and Water Research, C/ Jordi Girona, 18-26, 08034, Barcelona, Spain.

[2]Doctoral Program in Analytical Chemistry and Environmental Science, Dep. Of Chemical Engineering and Analytical Chemistry, University of Barcelona, Barcelona, Spain.

[3]VISAFELab Laboratory, Department of Preservation and Food Safety Technologies, IATA-CSIC, 46980 Valencia, Spain

[4] Department of Food Hygiene and Environmental Health, Faculty of Veterinary Medicine, University of Helsinki, P.O. 66 (Agnes Sjöbergin katu 2); 00014 University of Helsinki, Finland

[5]Agence Nationale de Sécurité Sanitaire de l'Alimentation, de l'Environnement et du Travail Maisons-Alfort (ANSES), France

***Corresponding author:** mlcqam@cid.csic.es , mfuqam@cid.csic.es

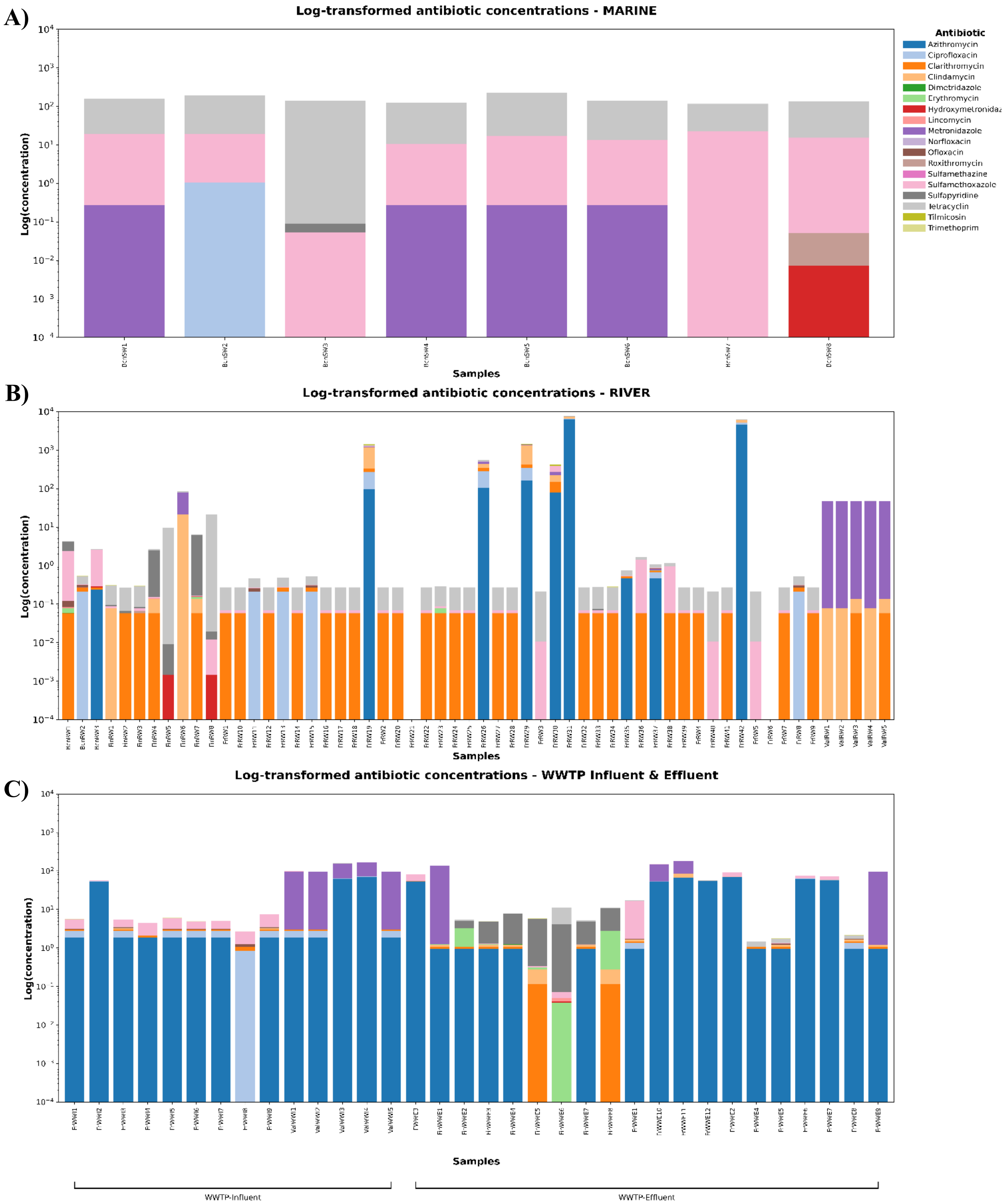

Figure S1. Antibiotic concentrations detected in sea (A), river (B), and near WWTPs (C) water samples. Sample codes starting with "Fr" belong to Paris, those starting with "Bcn" refer to Barcelona, those starting with "Fin" belong to Helsinki and those starting with "Val" were taken in València.

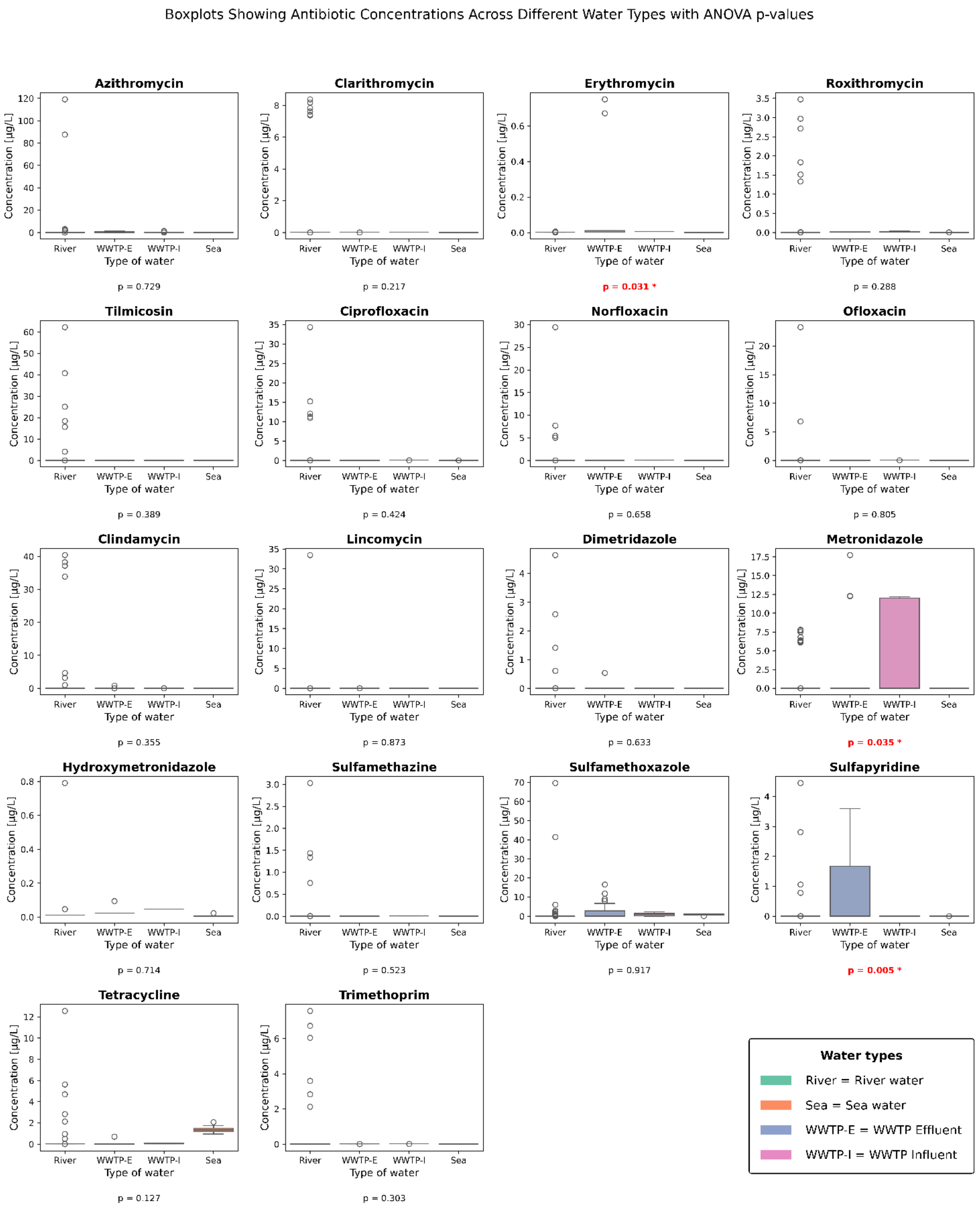

Figure S2. Boxplots showing antibiotic concentrations across different water types with ANOVA p-values (p-values in red* are statistically significant).

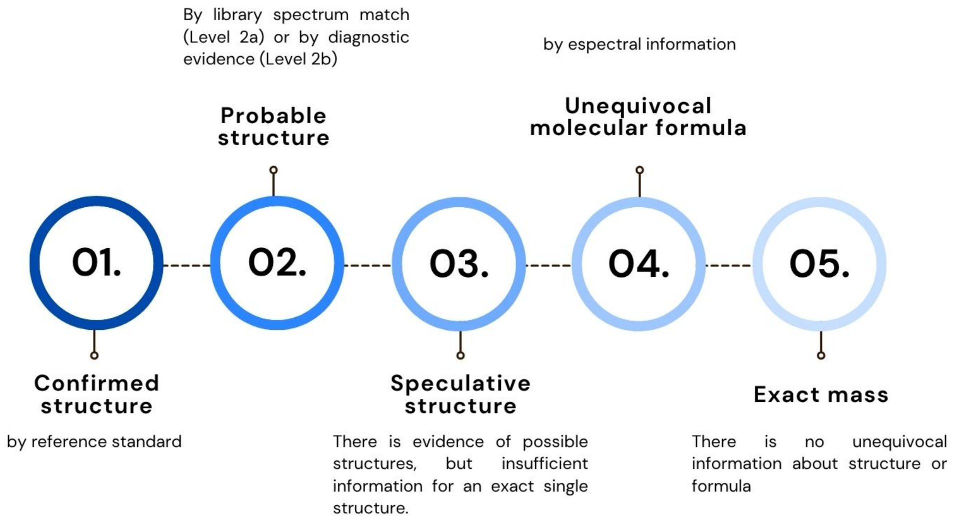

Figure S3. Confidence levels for the Suspect screening identification.

Table S1. Detection frequency and concentration levels of antibiotic drugs in rivers, sea and samples collected near WWTPs.

Compounds classification	Antibiotic	Detected frequency [%]					Concentration [µg/L]		
		River samples	Sea samples	WWTPs Samples		Total samples	Maximum	Minimum	Mean
				Influent	Effluent				
**Macrolides**	Azithromycin	9.09	0	11.11	52.94	16.00	119.10	1.01	14.17
	Clarithromycin	9.09	0	0	0	6.00	8.38	7.37	7.80
	Erythromycin	3.03	0	0	0	2.00	0.75	0.67	0.71
	Roxithromycin	9.09	0	0	0	6.00	3.48	1.33	2.31
	Tilmicosin	9.09	0	0	0	6.00	62.24	4.08	27.71
**Fluoroquinolones**	Ciprofloxacin	7.58	0	0	0	5.00	34.28	10.93	16.74
	Norfloxacin	6.06	0	0	0	4.00	29.43	5.00	11.88
	Ofloxacin	3.03	0	0	0	2.00	23.29	6.81	15.05
**Lincosamides**	Clindamycin	10.61	0	0	5.88	8.00	40.40	0.76	19.90
	Lincomycin	1.52	0	0	0	1.00	33.46	33.46	33.46
**Nitroimidazoles**	Dimetridazole	7.58	0	0	0	5.00	4.64	0.54	1.96
	Metronidazole	19.70	0	0	47.05	21.00	17.73	6.09	9.26
	Hydroxymetronidazole	1.52	0	0	0	1.00	0.79	0.79	0.79
**Sulfonamides**	Sulfamethazine	6.06	0	0	0	4.00	3.03	0.76	1.64
	Sulfamethoxazole	13.64	88.00	100.00	41.17	32.00	69.65	0.55	6.26
	Sulfapyridine	18.18	0	0	0	12.00	4.45	0.62	2.08
**Tetracyclines**	Tetracycline	12.12	100.00	0	0	16.00	12.57	0.50	2.58
**Diaminopyrimidine**	Trimethoprim	9.09	0	0	0	6.00	7.57	2.12	4.82
**Total number of samples**		66	8	9	17	100			

Table S2. Detailed concentrations (μg/L) of the analysed antibiotic.

SAMPLES	**Sample Concentration** [μg/L]																	
	Azithromycin	*Clarithromycin*	*Erythromycin*	*Roxithromycin*	*Tilmicosin*	*Ciprofloxacin*	*Norfloxacin*	*Ofloxacin*	*Clindamycin*	*Lincomycin*	*Dimetridazole*	*Metronidazole*	*Hydroxymetronidazole*	*Sulfamethazine*	*Sulfamethoxazole*	*Sulfapyridine*	*Tetracyclin*	*Trimethoprim*
**FinRW1**	ND	ND	ND	ND	ND	ND	ND	ND	<LOQ	ND	<LOQ	ND	ND	ND	<LOQ	<LOQ	<LOQ	<LOQ
**FinRW2**	ND	<LOQ	ND	ND	ND	ND	ND	ND	ND	ND	ND	ND	ND	ND	ND	<LOQ	<LOQ	ND
**FinRW3**	ND	<LOQ	ND	<LOQ	ND	ND	ND	ND	ND	ND	ND	ND	ND	ND	<LOQ	<LOQ	<LOQ	<LOQ
**FinRW4**	ND	<LOQ	ND	ND	ND	ND	ND	ND	<LOQ	<LOQ	ND	ND	ND	ND	<LOQ	1.0585	<LOQ	<LOQ
**FinWWE1**	<LOQ	<LOQ	<LOQ	<LOQ	ND	ND	ND	ND	<LOQ	ND	ND	17.7311	ND	ND	<LOQ	0.6225	ND	<LOQ
**FinWWE2**	<LOQ	<LOQ	0.6711	ND	ND	ND	ND	ND	ND	ND	ND	ND	ND	ND	ND	0.8319	<LOQ	ND
**FinWWE3**	<LOQ	<LOQ	<LOQ	<LOQ	ND	ND	ND	ND	<LOQ	ND	ND	ND	ND	ND	<LOQ	1.6673	ND	<LOQ
**FinWWE4**	<LOQ	<LOQ	ND	ND	ND	ND	ND	ND	<LOQ	ND	0.5363	ND	ND	ND	<LOQ	3.0646	ND	<LOQ
**FrRW1**	ND	<LOQ	ND	ND	ND	ND	ND	ND	ND	ND	ND	ND	ND	ND	<LOQ	ND	<LOQ	ND
**FrRW2**	ND	<LOQ	ND	ND	ND	ND	ND	ND	ND	ND	ND	ND	ND	ND	<LOQ	ND	<LOQ	ND
**FrRW3**	ND	ND	ND	ND	ND	ND	ND	ND	ND	ND	ND	ND	ND	ND	<LOQ	ND	<LOQ	ND
**FrRW4**	ND	<LOQ	ND	ND	ND	ND	ND	ND	ND	ND	ND	ND	ND	ND	<LOQ	ND	<LOQ	ND
**FrRW5**	ND	ND	ND	ND	ND	ND	ND	ND	ND	ND	ND	ND	ND	ND	<LOQ	ND	<LOQ	ND
**FrRW6**	ND	ND	ND	ND	ND	ND	ND	ND	ND	ND	ND	ND	ND	ND	ND	ND	ND	ND
**FrRW7**	ND	<LOQ	ND	ND	ND	ND	ND	ND	ND	ND	ND	ND	ND	ND	<LOQ	ND	<LOQ	ND
**FrRW8**	ND	<LOQ	ND	ND	ND	<LOQ	ND	<LOQ	ND	ND	ND	ND	ND	ND	<LOQ	ND	<LOQ	ND
**FrRW9**	ND	<LOQ	ND	ND	ND	ND	ND	ND	ND	ND	ND	ND	ND	ND	<LOQ	ND	<LOQ	ND
**FrRW10**	ND	<LOQ	ND	ND	ND	ND	ND	ND	ND	ND	ND	ND	ND	ND	<LOQ	ND	<LOQ	ND

SAMPLES	Sample Concentration [µg/L]																	
	Azithromycin	*Clarithromycin*	*Erythromycin*	*Roxithromycin*	*Tilmicosin*	*Ciprofloxacin*	*Norfloxacin*	*Ofloxacin*	*Clindamycin*	*Lincomycin*	*Dimetridazole*	*Metronidazole*	*Hydroxymetronidazole*	*Sulfamethazine*	*Sulfamethoxazole*	*Sulfapyridine*	*Tetracyclin*	*Trimethoprim*
**FrRW11**	ND	ND	ND	ND	ND	<LOQ	ND	<LOQ	ND	<LOQ	ND	ND	ND	ND	<LOQ	ND	<LOQ	ND
**FrRW12**	ND	<LOQ	ND	ND	ND	ND	ND	ND	ND	ND	ND	ND	ND	ND	<LOQ	ND	<LOQ	ND
**FrRW13**	ND	<LOQ	ND	ND	ND	<LOQ	ND	ND	ND	ND	ND	ND	ND	ND	<LOQ	ND	<LOQ	ND
**FrRW14**	ND	<LOQ	ND	ND	ND	ND	ND	ND	ND	ND	ND	ND	ND	ND	<LOQ	ND	<LOQ	ND
**FrRW15**	ND	<LOQ	ND	ND	ND	<LOQ	ND	<LOQ	ND	ND	ND	ND	ND	ND	<LOQ	ND	<LOQ	ND
**FrRW16**	ND	<LOQ	ND	ND	ND	ND	ND	ND	ND	ND	ND	ND	ND	ND	<LOQ	ND	<LOQ	ND
**FrRW17**	ND	<LOQ	ND	ND	ND	ND	ND	ND	ND	ND	ND	ND	ND	ND	<LOQ	ND	<LOQ	ND
**FrRW18**	ND	<LOQ	ND	ND	ND	ND	ND	ND	ND	ND	ND	ND	ND	ND	<LOQ	ND	<LOQ	ND
**FrRW19**	1.8394	7.4083	<LOQ	3.4761	62.2395	10.9275	ND	<LOQ	37.1772	ND	1.4111	6.7944	<LOQ	<LOQ	41.3834	ND	5.6228	7.5746
**FrRW20**	ND	<LOQ	ND	ND	ND	ND	ND	ND	ND	ND	ND	ND	ND	ND	<LOQ	ND	<LOQ	ND
**FrRW21**	ND	ND	ND	ND	ND	ND	ND	ND	ND	ND	ND	ND	ND	ND	ND	ND	ND	ND
**FrRW22**	ND	<LOQ	ND	ND	ND	ND	ND	ND	ND	ND	ND	ND	ND	ND	<LOQ	ND	<LOQ	ND
**FrRW23**	ND	<LOQ	<LOQ	ND	ND	ND	ND	ND	ND	ND	ND	ND	ND	ND	<LOQ	ND	<LOQ	ND
**FrRW24**	ND	<LOQ	ND	ND	ND	ND	ND	ND	ND	ND	ND	ND	ND	ND	<LOQ	ND	<LOQ	ND
**FrRW25**	ND	<LOQ	ND	ND	ND	ND	ND	ND	ND	ND	ND	ND	ND	<LOQ	<LOQ	ND	<LOQ	ND
**FrRW26**	1.9831	7.3738	<LOQ	1.8339	4.0841	11.2479	5.0024	<LOQ	4.6303	<LOQ	0.6066	6.3206	ND	1.3374	2.1845	<LOQ	4.7004	3.5951
**FrRW27**	ND	<LOQ	ND	ND	ND	ND	ND	ND	ND	ND	ND	ND	ND	ND	<LOQ	ND	<LOQ	ND
**FrRW28**	ND	<LOQ	ND	ND	ND	ND	ND	ND	ND	ND	ND	ND	ND	ND	<LOQ	ND	<LOQ	ND
**FrRW29**	3.0977	8.3777	<LOQ	1.3322	25.0848	12.0345	5.4195	<LOQ	40.4045	<LOQ	<LOQ	6.3919	ND	<LOQ	<LOQ	ND	ND	2.8327

SAMPLES	Sample Concentration [µg/L]																	
	Azithromycin	*Clarithromycin*	*Erythromycin*	*Roxithromycin*	*Tilmicosin*	*Ciprofloxacin*	*Norfloxacin*	*Ofloxacin*	*Clindamycin*	*Lincomycin*	*Dimetridazole*	*Metronidazole*	*Hydroxymetronidazole*	*Sulfamethazine*	*Sulfamethoxazole*	*Sulfapyridine*	*Tetracyclin*	*Trimethoprim*
**FrRW30**	1.5282	8.1759	<LOQ	1.5140	40.7782	ND	ND	ND	3.1650	<LOQ	<LOQ	6.1434	ND	0.7558	69.6497	<LOQ	ND	2.1219
**FrRW31**	119.0977	7.6110	<LOQ	2.9717	18.3751	15.2142	7.6825	6.8120	38.2576	<LOQ	2.5804	7.4957	<LOQ	3.0316	5.9993	4.4533	2.8301	6.7267
**FrRW32**	ND	<LOQ	ND	ND	ND	ND	ND	ND	ND	ND	ND	ND	ND	ND	<LOQ	ND	<LOQ	ND
**FrRW33**	ND	<LOQ	ND	ND	ND	ND	ND	ND	ND	ND	ND	ND	ND	ND	<LOQ	<LOQ	<LOQ	ND
**FrRW34**	ND	<LOQ	ND	ND	ND	ND	ND	ND	ND	ND	ND	ND	ND	ND	<LOQ	ND	<LOQ	<LOQ
**FrRW35**	<LOQ	<LOQ	ND	ND	ND	ND	ND	ND	ND	ND	<LOQ	ND	ND	ND	<LOQ	ND	<LOQ	<LOQ
**FrRW36**	ND	<LOQ	ND	ND	ND	ND	ND	ND	ND	ND	ND	ND	ND	ND	0.8367	ND	<LOQ	<LOQ
**FrRW37**	<LOQ	<LOQ	<LOQ	<LOQ	ND	<LOQ	ND	<LOQ	ND	ND	<LOQ	<LOQ	ND	ND	<LOQ	ND	<LOQ	<LOQ
**FrRW38**	ND	<LOQ	ND	ND	ND	ND	ND	ND	ND	ND	ND	ND	ND	ND	0.5450	ND	<LOQ	ND
**FrRW39**	ND	<LOQ	ND	ND	ND	ND	ND	ND	ND	ND	ND	ND	ND	ND	<LOQ	ND	<LOQ	ND
**FrRW40**	ND	ND	ND	ND	ND	ND	ND	ND	ND	ND	ND	ND	ND	ND	<LOQ	ND	<LOQ	ND
**FrRW41**	ND	<LOQ	ND	ND	ND	ND	ND	ND	ND	ND	ND	ND	ND	ND	<LOQ	ND	<LOQ	ND
**FrRW42**	87.5741	7.8316	<LOQ	2.7127	15.6826	34.2839	29.4321	23.2856	33.8861	33.4578	4.6416	7.7961	<LOQ	1.4331	2.8197	ND	12.5657	6.0443
**FrWWE1**	<LOQ	<LOQ	ND	<LOQ	ND	<LOQ	ND	<LOQ	<LOQ	ND	ND	ND	ND	ND	8.9733	ND	<LOQ	<LOQ
**FrWWE2**	1.3347	<LOQ	<LOQ	<LOQ	ND	<LOQ	ND	<LOQ	<LOQ	ND	ND	ND	ND	ND	11.8056	ND	<LOQ	<LOQ
**FWWE3**	1.0098	<LOQ	<LOQ	ND	ND	<LOQ	ND	<LOQ	<LOQ	ND	ND	ND	ND	ND	16.5496	ND	<LOQ	<LOQ
**FrWWE4**	<LOQ	<LOQ	ND	ND	ND	ND	ND	ND	ND	ND	ND	ND	ND	ND	ND	ND	<LOQ	ND
**FrWWE5**	<LOQ	<LOQ	ND	<LOQ	ND	ND	ND	<LOQ	<LOQ	ND	ND	ND	ND	ND	<LOQ	ND	<LOQ	<LOQ
**FrWWE6**	1.1933	<LOQ	ND	<LOQ	ND	<LOQ	ND	<LOQ	<LOQ	ND	ND	ND	ND	ND	6.6485	ND	<LOQ	<LOQ

SAMPLES	Sample Concentration [µg/L]																	
	Azithromycin	*Clarithromycin*	*Erythromycin*	*Roxithromycin*	*Tilmicosin*	*Ciprofloxacin*	*Norfloxacin*	*Ofloxacin*	*Clindamycin*	*Lincomycin*	*Dimetridazole*	*Metronidazole*	*Hydroxymetronidazole*	*Sulfamethazine*	*Sulfamethoxazole*	*Sulfapyridine*	*Tetracyclin*	*Trimethoprim*
**FrWWE7**	1.1018	<LOQ	ND	ND	ND	<LOQ	ND	<LOQ	<LOQ	ND	ND	ND	ND	ND	7.9413	ND	<LOQ	<LOQ
**FrWWE8**	<LOQ	<LOQ	ND	ND	ND	<LOQ	ND	<LOQ	<LOQ	ND	ND	ND	ND	ND	ND	ND	<LOQ	<LOQ
**BcnRW1**	ND	<LOQ	<LOQ	ND	ND	ND	ND	<LOQ	ND	<LOQ	<LOQ	ND	<LOQ	ND	1.3830	0.7816	<LOQ	<LOQ
**BcnRW2**	ND	<LOQ	ND	ND	ND	<LOQ	ND	<LOQ	ND	<LOQ	<LOQ	ND	ND	ND	<LOQ	ND	<LOQ	<LOQ
**BcnSW1**	ND	ND	ND	ND	ND	ND	ND	ND	ND	ND	ND	<LOQ	ND	ND	1.1204	ND	1.4150	ND
**FinRW5**	ND	ND	ND	ND	ND	ND	ND	ND	ND	ND	ND	ND	<LOQ	ND	ND	<LOQ	0.9567	ND
**FinRW6**	ND	ND	ND	ND	ND	ND	ND	ND	0.9525	ND	ND	7.6985	<LOQ	ND	ND	<LOQ	0.5015	ND
**FinRW7**	ND	<LOQ	<LOQ	ND	ND	ND	ND	ND	<LOQ	ND	ND	ND	<LOQ	ND	<LOQ	2.8133	<LOQ	<LOQ
**FinRW8**	ND	ND	ND	ND	ND	ND	ND	ND	ND	ND	ND	ND	<LOQ	ND	<LOQ	<LOQ	2.1337	ND
**FinWWE5**	ND	<LOQ	<LOQ	ND	ND	ND	ND	ND	<LOQ	ND	ND	ND	<LOQ	ND	<LOQ	2.5045	ND	<LOQ
**FinWWE6**	ND	ND	<LOQ	ND	ND	ND	ND	ND	ND	<LOQ	ND	ND	<LOQ	ND	<LOQ	1.8380	0.7023	<LOQ
**FinWWE7**	<LOQ	<LOQ	ND	<LOQ	ND	ND	ND	ND	<LOQ	ND	ND	ND	<LOQ	ND	<LOQ	1.6700	<LOQ	<LOQ
**FinWWE8**	ND	<LOQ	0.7495	ND	ND	ND	ND	ND	<LOQ	ND	ND	ND	<LOQ	ND	<LOQ	3.5967	<LOQ	<LOQ
**BcnSW2**	ND	ND	ND	ND	ND	<LOQ	ND	ND	ND	ND	ND	ND	ND	ND	1.0830	ND	1.7420	ND
**BcnSW3**	ND	ND	ND	ND	ND	ND	ND	ND	ND	ND	ND	ND	ND	ND	<LOQ	<LOQ	1.4108	ND
**BcnRW3**	<LOQ	<LOQ	ND	ND	<LOQ	ND	ND	ND	ND	ND	ND	ND	0.7907	ND	1.4074	<LOQ	<LOQ	ND
**BcnSW4**	ND	ND	ND	ND	ND	ND	ND	ND	ND	ND	ND	<LOQ	ND	ND	0.6082	ND	1.1571	ND
**BcnSW5**	ND	ND	ND	ND	ND	ND	ND	ND	ND	ND	ND	<LOQ	ND	ND	0.9977	ND	2.0740	ND
**BcnSW6**	ND	ND	ND	ND	ND	ND	ND	ND	ND	ND	ND	<LOQ	ND	ND	0.7963	ND	1.2668	ND

SAMPLES	**Sample Concentration** [µg/L]																	
	Azithromycin	*Clarithromycin*	*Erythromycin*	*Roxithromycin*	*Tilmicosin*	*Ciprofloxacin*	*Norfloxacin*	*Ofloxacin*	*Clindamycin*	*Lincomycin*	*Dimetridazole*	*Metronidazole*	*Hydroxymetronidazole*	*Sulfamethazine*	*Sulfamethoxazole*	*Sulfapyridine*	*Tetracyclin*	*Trimethoprim*
**BcnSW7**	ND	ND	ND	ND	ND	ND	ND	ND	ND	ND	ND	ND	ND	ND	1.3310	ND	0.9550	ND
**BcnSW8**	ND	ND	ND	<LOQ	ND	ND	ND	ND	ND	ND	ND	ND	<LOQ	ND	0.9255	ND	1.2151	ND
**FrWWI1**	<LOQ	<LOQ	ND	ND	ND	<LOQ	ND	<LOQ	ND	ND	ND	ND	ND	ND	1.4702	ND	ND	<LOQ
**FrWWI2**	1.0200	<LOQ	ND	<LOQ	ND	<LOQ	ND	<LOQ	ND	ND	ND	ND	ND	ND	0.8949	ND	ND	<LOQ
**FrWWI3**	<LOQ	<LOQ	ND	<LOQ	ND	<LOQ	ND	<LOQ	<LOQ	ND	ND	ND	ND	ND	1.1683	ND	ND	<LOQ
**FrWWI4**	<LOQ	<LOQ	ND	ND	ND	ND	ND	ND	ND	ND	ND	ND	ND	ND	1.4019	ND	ND	<LOQ
**FrWWI5**	<LOQ	<LOQ	ND	ND	ND	<LOQ	ND	<LOQ	ND	ND	ND	ND	ND	ND	1.6924	<LOQ	ND	<LOQ
**FrWWI6**	<LOQ	<LOQ	ND	ND	ND	<LOQ	ND	<LOQ	ND	ND	ND	ND	ND	ND	1.0601	ND	ND	<LOQ
**FrWWI7**	<LOQ	<LOQ	ND	<LOQ	ND	<LOQ	ND	<LOQ	ND	ND	ND	ND	ND	ND	1.1512	ND	ND	<LOQ
**FrWWI8**	ND	<LOQ	ND	ND	ND	<LOQ	ND	<LOQ	ND	ND	ND	ND	ND	ND	0.8607	ND	ND	<LOQ
**FrWWI9**	<LOQ	<LOQ	ND	<LOQ	ND	<LOQ	ND	<LOQ	<LOQ	ND	ND	ND	ND	ND	2.3987	<LOQ	ND	<LOQ
**ValWWI1**	<LOQ	<LOQ	ND	ND	ND	<LOQ	ND	<LOQ	ND	ND	ND	12.1714	ND	ND	1.6693	ND	<LOQ	ND
**ValWWI2**	<LOQ	<LOQ	ND	ND	ND	<LOQ	ND	<LOQ	ND	ND	ND	12.0325	ND	ND	<LOQ	ND	<LOQ	<LOQ
**ValWWI3**	1.1965	<LOQ	ND	ND	ND	<LOQ	ND	<LOQ	ND	ND	ND	12.0341	ND	ND	<LOQ	<LOQ	<LOQ	<LOQ
**ValWWI4**	1.3469	<LOQ	ND	ND	ND	<LOQ	ND	<LOQ	ND	ND	ND	12.0746	ND	ND	<LOQ	<LOQ	<LOQ	<LOQ
**ValWWI5**	<LOQ	<LOQ	ND	ND	ND	<LOQ	ND	<LOQ	ND	ND	ND	12.1352	ND	ND	<LOQ	<LOQ	<LOQ	<LOQ
**ValRW1**	ND	ND	ND	ND	ND	ND	ND	ND	<LOQ	ND	ND	6.1198	ND	ND	<LOQ	ND	<LOQ	ND
**ValRW2**	ND	ND	ND	ND	ND	ND	ND	ND	<LOQ	ND	ND	6.1349	ND	ND	<LOQ	ND	<LOQ	ND
**ValRW3**	ND	<LOQ	ND	ND	ND	ND	ND	ND	<LOQ	ND	ND	6.1680	ND	ND	<LOQ	ND	<LOQ	ND

SAMPLES	Sample Concentration [µg/L]																	
	Azithromycin	*Clarithromycin*	*Erythromycin*	*Roxithromycin*	*Tilmicosin*	*Ciprofloxacin*	*Norfloxacin*	*Ofloxacin*	*Clindamycin*	*Lincomycin*	*Dimetridazole*	*Metronidazole*	*Hydroxymetronidazole*	*Sulfamethazine*	*Sulfamethoxazole*	*Sulfapyridine*	*Tetracyclin*	*Trimethoprim*
**ValRW4**	ND	ND	ND	ND	ND	ND	ND	ND	<LOQ	ND	ND	6.2475	ND	ND	<LOQ	ND	<LOQ	ND
**ValRW5**	ND	<LOQ	ND	ND	ND	ND	ND	<LOQ	<LOQ	ND	ND	6.0857	ND	ND	<LOQ	ND	<LOQ	ND
**FrWWE9**	<LOQ	<LOQ	ND	<LOQ	ND	ND	ND	ND	<LOQ	ND	ND	12.2810	ND	ND	<LOQ	<LOQ	<LOQ	ND
**FrWWE10**	1.0126	<LOQ	ND	<LOQ	ND	<LOQ	ND	<LOQ	ND	ND	ND	12.3129	ND	ND	1.5269	<LOQ	ND	<LOQ
**FrWWE11**	1.2818	<LOQ	ND	<LOQ	ND	<LOQ	ND	<LOQ	0.7624	ND	ND	12.2596	ND	ND	<LOQ	<LOQ	ND	<LOQ
**FrWWE12**	1.0552	<LOQ	ND	<LOQ	ND	ND	ND	<LOQ	<LOQ	ND	ND	ND	ND	ND	<LOQ	ND	<LOQ	<LOQ

Table S3. Calculated limits of detection (LOD) and quantification (LOQ) for each antibiotic in each water matrix.

Antibiotic	LOD [µg/L]				LOQ [µg/L]			
	Seawater	River water	WWTP Influent	WWTP Effluent	Seawater	River water	WWTP Influent	WWTP Effluent
Azithromycin	0.0011	0.0023	0.0090	0.0045	0.0045	0.0090	0.0360	0.0180
Clarithromycin	0.0009	0.0018	0.0070	0.0035	0.0035	0.0070	0.0280	0.0140
Erythromycin	0.0007	0.0014	0.0057	0.0029	0.0029	0.0057	0.0229	0.0115
Roxithromycin	0.0011	0.0022	0.0087	0.0043	0.0043	0.0087	0.0347	0.0173
Tilmicosin	0.0013	0.0026	0.0105	0.0052	0.0052	0.0105	0.0419	0.0209
Ciprofloxacin	0.0017	0.0034	0.0135	0.0068	0.0068	0.0135	0.0541	0.0271
Norfloxacin	0.0051	0.0103	0.0410	0.0205	0.0205	0.0410	0.1640	0.0820
Ofloxacin	0.0025	0.0049	0.0197	0.0098	0.0098	0.0197	0.0787	0.0393
Clindamycin	0.0004	0.0009	0.0035	0.0017	0.0017	0.0035	0.0139	0.0069
Lincomycin	0.0011	0.0021	0.0085	0.0043	0.0043	0.0085	0.0341	0.0171
Dimetridazole	0.0003	0.0005	0.0021	0.0010	0.0010	0.0021	0.0083	0.0041
Metronidazole	0.0009	0.0018	0.0072	0.0036	0.0036	0.0072	0.0288	0.0144
Hydroxymetronidazole	0.0059	0.0118	0.0473	0.0237	0.0237	0.0473	0.1893	0.0947
Sulfamethazine	0.0006	0.0013	0.0050	0.0025	0.0025	0.0050	0.0200	0.0100
Sulfamethoxazole	0.0008	0.0016	0.0063	0.0032	0.0032	0.0063	0.0253	0.0127
Sulfapyridine	0.0004	0.0009	0.0035	0.0018	0.0018	0.0035	0.0141	0.0071
Tetracycline	0.0026	0.0051	0.0205	0.0103	0.0103	0.0205	0.0821	0.0411
Trimethoprim	0.0008	0.0016	0.0065	0.0032	0.0032	0.0065	0.0259	0.0129

Table S4. Summary of predicted no-effect concentrations (PNECs), octanol-water partition coefficients for the selected compounds. Values provided by EPA United States Environmental Protection Agency database, and Norman databases.

Antibiotic	PNEC [µg/L]		Log (Kow) experimental	Log (Kow)*	LogD (pH = 7.4)*
	Seawater	River water			
Azithromycin	0.0019	0.0190	4.0	3.7	2.7
Clarithromycin	0.0130	0.1200	2.9	3.1	2.8
Erythromycin	0.0500	0.3000	2.9	2.8	2.4
Roxithromycin	0.1000	1.0000	3.4	-	-
Tilmicosin	0.1000	1.0000	3.8	3.9	0.8
Ciprofloxacin	0.0064	0.0640	0.3	0.0	-1.5
Norfloxacin	0.0500	0.5000	0.1	-0.8	-6.6
Ofloxacin	0.0500	0.5000	0.2	-0.1	-4.4
Clindamycin	0.0044	0.0440	2.1	2.1	2.0
Lincomycin	0.2000	2.0000	0.4	0.4	0.2
Dimetridazole	2.9500	29.5000	0.3	0.4	0.4
Metronidazole	0.0130	0.1300	0.0	0.0	0.0
Hydroxymetronidazole	3.2800	32.8000	-	-0.8	-0.8
Sulfamethazine	3.0000	30.0000	0.4	0.3	0.1
Sulfamethoxazole	0.0600	0.6000	0.9	0.8	-0.5
Sulfapyridine	0.0460	0.4600	0.4	0.4	-0.5
Tetracycline	0.0100	0.1000	-1.2	-1.0	-2.8
Trimethoprim	10.0000	0.5000	0.9	1.0	0.9

*Predicted average values

Table S5. Detailed Hazard Quotient (HQ) of the analysed antibiotic in each water sample, with colour coding indicating risk levels (green, yellow, orange, red – from lowest to highest).

SAMPLES	Hazard Quotient																	
	Azithromycin	*Clarithromycin*	*Erythromycin*	*Roxithromycin*	*Tilmicosin*	*Ciprofloxacin*	*Norfloxacin*	*Ofloxacin*	*Clindamycin*	*Lincomycin*	*Dimetridazole*	*Metronidazole*	*Hydroxymetronidazole*	*Sulfamethazine*	*Sulfamethoxazole*	*Sulfapyridine*	*Tetracyclin*	*Trimethoprim*
**FinRW1**	0.00	0.00	0.00	0.00	0.00	0.00	0.00	0.00	0.08	0.00	0.00	0.00	0.00	0.00	0.01	0.01	0.21	0.01
**FinRW2**	0.00	0.06	0.00	0.00	0.00	0.00	0.00	0.00	0.00	0.00	0.00	0.00	0.00	0.00	0.00	0.01	0.21	0.00
**FinRW3**	0.00	0.06	0.00	0.01	0.00	0.00	0.00	0.00	0.00	0.00	0.00	0.00	0.00	0.00	0.01	0.01	0.21	0.01
**FinRW4**	0.00	0.06	0.00	0.00	0.00	0.00	0.00	0.00	0.08	0.00	0.00	0.00	0.00	0.00	0.01	2.30	0.21	0.01
**FinWWE1**	0.95	0.12	0.04	0.02	0.00	0.00	0.00	0.00	0.16	0.00	0.00	136.39	0.00	0.00	0.02	1.35	0.00	0.03
**FinWWE2**	0.95	0.12	2.24	0.00	0.00	0.00	0.00	0.00	0.00	0.00	0.00	0.00	0.00	0.00	0.00	1.81	0.41	0.00
**FinWWE3**	0.95	0.12	0.04	0.02	0.00	0.00	0.00	0.00	0.16	0.00	0.00	0.00	0.00	0.00	0.02	3.62	0.00	0.03
**FinWWE4**	0.95	0.12	0.00	0.00	0.00	0.00	0.00	0.00	0.16	0.00	0.02	0.00	0.00	0.00	0.02	6.66	0.00	0.03
**FrRW1**	0.00	0.06	0.00	0.00	0.00	0.00	0.00	0.00	0.00	0.00	0.00	0.00	0.00	0.00	0.01	0.00	0.21	0.00
**FrRW2**	0.00	0.06	0.00	0.00	0.00	0.00	0.00	0.00	0.00	0.00	0.00	0.00	0.00	0.00	0.01	0.00	0.21	0.00
**FrRW3**	0.00	0.00	0.00	0.00	0.00	0.00	0.00	0.00	0.00	0.00	0.00	0.00	0.00	0.00	0.01	0.00	0.21	0.00
**FrRW4**	0.00	0.06	0.00	0.00	0.00	0.00	0.00	0.00	0.00	0.00	0.00	0.00	0.00	0.00	0.01	0.00	0.21	0.00
**FrRW5**	0.00	0.00	0.00	0.00	0.00	0.00	0.00	0.00	0.00	0.00	0.00	0.00	0.00	0.00	0.01	0.00	0.21	0.00
**FrRW6**	0.00	0.00	0.00	0.00	0.00	0.00	0.00	0.00	0.00	0.00	0.00	0.00	0.00	0.00	0.00	0.00	0.00	0.00
**FrRW7**	0.00	0.06	0.00	0.00	0.00	0.00	0.00	0.00	0.00	0.00	0.00	0.00	0.00	0.00	0.01	0.00	0.21	0.00
**FrRW8**	0.00	0.06	0.00	0.00	0.00	0.21	0.00	0.04	0.00	0.00	0.00	0.00	0.00	0.00	0.01	0.00	0.21	0.00
**FrRW9**	0.00	0.06	0.00	0.00	0.00	0.00	0.00	0.00	0.00	0.00	0.00	0.00	0.00	0.00	0.01	0.00	0.21	0.00

**FrRW10**	0.00	0.06	0.00	0.00	0.00	0.00	0.00	0.00	0.00	0.00	0.00	0.00	0.00	0.00	0.01	0.00	0.21	0.00
**FrRW11**	0.00	0.00	0.00	0.00	0.00	0.21	0.00	0.04	0.00	0.00	0.00	0.00	0.00	0.00	0.01	0.00	0.21	0.00
**FrRW12**	0.00	0.06	0.00	0.00	0.00	0.00	0.00	0.00	0.00	0.00	0.00	0.00	0.00	0.00	0.01	0.00	0.21	0.00
**FrRW13**	0.00	0.06	0.00	0.00	0.00	0.21	0.00	0.00	0.00	0.00	0.00	0.00	0.00	0.00	0.01	0.00	0.21	0.00
**FrRW14**	0.00	0.06	0.00	0.00	0.00	0.00	0.00	0.00	0.00	0.00	0.00	0.00	0.00	0.00	0.01	0.00	0.21	0.00
**FrRW15**	0.00	0.06	0.00	0.00	0.00	0.21	0.00	0.04	0.00	0.00	0.00	0.00	0.00	0.00	0.01	0.00	0.21	0.00
**FrRW16**	0.00	0.06	0.00	0.00	0.00	0.00	0.00	0.00	0.00	0.00	0.00	0.00	0.00	0.00	0.01	0.00	0.21	0.00
**FrRW17**	0.00	0.06	0.00	0.00	0.00	0.00	0.00	0.00	0.00	0.00	0.00	0.00	0.00	0.00	0.01	0.00	0.21	0.00
**FrRW18**	0.00	0.06	0.00	0.00	0.00	0.00	0.00	0.00	0.00	0.00	0.00	0.00	0.00	0.00	0.01	0.00	0.21	0.00
**FrRW19**	96.81	61.74	0.02	3.48	62.24	170.74	0.00	0.04	844.94	0.00	0.05	52.26	0.00	0.00	68.97	0.00	56.23	15.15
**FrRW20**	0.00	0.06	0.00	0.00	0.00	0.00	0.00	0.00	0.00	0.00	0.00	0.00	0.00	0.00	0.01	0.00	0.21	0.00
**FrRW21**	0.00	0.00	0.00	0.00	0.00	0.00	0.00	0.00	0.00	0.00	0.00	0.00	0.00	0.00	0.00	0.00	0.00	0.00
**FrRW22**	0.00	0.06	0.00	0.00	0.00	0.00	0.00	0.00	0.00	0.00	0.00	0.00	0.00	0.00	0.01	0.00	0.21	0.00
**FrRW23**	0.00	0.06	0.02	0.00	0.00	0.00	0.00	0.00	0.00	0.00	0.00	0.00	0.00	0.00	0.01	0.00	0.21	0.00
**FrRW24**	0.00	0.06	0.00	0.00	0.00	0.00	0.00	0.00	0.00	0.00	0.00	0.00	0.00	0.00	0.01	0.00	0.21	0.00
**FrRW25**	0.00	0.06	0.00	0.00	0.00	0.00	0.00	0.00	0.00	0.00	0.00	0.00	0.00	0.00	0.01	0.00	0.21	0.00
**FrRW26**	104.37	61.45	0.02	1.83	4.08	175.75	10.00	0.04	105.23	0.00	0.02	48.62	0.00	0.04	3.64	0.01	47.00	7.19
**FrRW27**	0.00	0.06	0.00	0.00	0.00	0.00	0.00	0.00	0.00	0.00	0.00	0.00	0.00	0.00	0.01	0.00	0.21	0.00
**FrRW28**	0.00	0.06	0.00	0.00	0.00	0.00	0.00	0.00	0.00	0.00	0.00	0.00	0.00	0.00	0.01	0.00	0.21	0.00
**FrRW29**	163.04	69.81	0.02	1.33	25.08	188.04	10.84	0.04	918.28	0.00	0.00	49.17	0.00	0.00	0.01	0.00	0.00	5.67
**FrRW30**	80.43	68.13	0.02	1.51	40.78	0.00	0.00	0.00	71.93	0.00	0.00	47.26	0.00	0.03	116.08	0.01	0.00	4.24
**FrRW31**	6268.30	63.42	0.02	2.97	18.38	237.72	15.37	13.62	869.49	0.00	0.09	57.66	0.00	0.10	10.00	9.68	28.30	13.45
**FrRW32**	0.00	0.06	0.00	0.00	0.00	0.00	0.00	0.00	0.00	0.00	0.00	0.00	0.00	0.00	0.01	0.00	0.21	0.00
**FrRW33**	0.00	0.06	0.00	0.00	0.00	0.00	0.00	0.00	0.00	0.00	0.00	0.00	0.00	0.00	0.01	0.01	0.21	0.00
**FrRW34**	0.00	0.06	0.00	0.00	0.00	0.00	0.00	0.00	0.00	0.00	0.00	0.00	0.00	0.00	0.01	0.00	0.21	0.01

**FrRW35**	0.47	0.06	0.00	0.00	0.00	0.00	0.00	0.00	0.00	0.00	0.00	0.00	0.00	0.00	0.01	0.00	0.21	0.01
**FrRW36**	0.00	0.06	0.00	0.00	0.00	0.00	0.00	0.00	0.00	0.00	0.00	0.00	0.00	0.00	1.39	0.00	0.21	0.01
**FrRW37**	0.47	0.06	0.02	0.01	0.00	0.21	0.00	0.04	0.00	0.00	0.00	0.06	0.00	0.00	0.01	0.00	0.21	0.01
**FrRW38**	0.00	0.06	0.00	0.00	0.00	0.00	0.00	0.00	0.00	0.00	0.00	0.00	0.00	0.00	0.91	0.00	0.21	0.00
**FrRW39**	0.00	0.06	0.00	0.00	0.00	0.00	0.00	0.00	0.00	0.00	0.00	0.00	0.00	0.00	0.01	0.00	0.21	0.00
**FrRW40**	0.00	0.00	0.00	0.00	0.00	0.00	0.00	0.00	0.00	0.00	0.00	0.00	0.00	0.00	0.01	0.00	0.21	0.00
**FrRW41**	0.00	0.06	0.00	0.00	0.00	0.00	0.00	0.00	0.00	0.00	0.00	0.00	0.00	0.00	0.01	0.00	0.21	0.00
**FrRW42**	4609.16	65.26	0.02	2.71	15.68	535.69	58.86	46.57	770.14	16.73	0.16	59.97	0.00	0.05	4.70	0.00	125.66	12.09
**FrWWE1**	0.95	0.12	0.00	0.02	0.00	0.42	0.00	0.08	0.16	0.00	0.00	0.00	0.00	0.00	14.96	0.00	0.41	0.03
**FrWWE2**	70.25	0.12	0.04	0.02	0.00	0.42	0.00	0.08	0.16	0.00	0.00	0.00	0.00	0.00	19.68	0.00	0.41	0.03
**FWWE3**	53.15	0.12	0.04	0.00	0.00	0.42	0.00	0.08	0.16	0.00	0.00	0.00	0.00	0.00	27.58	0.00	0.41	0.03
**FrWWE4**	0.95	0.12	0.00	0.00	0.00	0.00	0.00	0.00	0.00	0.00	0.00	0.00	0.00	0.00	0.00	0.00	0.41	0.00
**FrWWE5**	0.95	0.12	0.00	0.02	0.00	0.00	0.00	0.08	0.16	0.00	0.00	0.00	0.00	0.00	0.02	0.00	0.41	0.03
**FrWWE6**	62.81	0.12	0.00	0.02	0.00	0.42	0.00	0.08	0.16	0.00	0.00	0.00	0.00	0.00	11.08	0.00	0.41	0.03
**FrWWE7**	57.99	0.12	0.00	0.00	0.00	0.42	0.00	0.08	0.16	0.00	0.00	0.00	0.00	0.00	13.24	0.00	0.41	0.03
**FrWWE8**	0.95	0.12	0.00	0.00	0.00	0.42	0.00	0.08	0.16	0.00	0.00	0.00	0.00	0.00	0.00	0.00	0.41	0.03
**BcnRW1**	0.00	0.06	0.02	0.00	0.00	0.00	0.00	0.04	0.00	0.00	0.00	0.00	0.00	0.00	2.31	1.70	0.21	0.01
**BcnRW2**	0.00	0.06	0.00	0.00	0.00	0.21	0.00	0.04	0.00	0.00	0.00	0.00	0.00	0.00	0.01	0.00	0.21	0.01
**BcnSW1**	0.00	0.00	0.00	0.00	0.00	0.00	0.00	0.00	0.00	0.00	0.00	0.28	0.00	0.00	18.67	0.00	141.50	0.00
**FinRW5**	0.00	0.00	0.00	0.00	0.00	0.00	0.00	0.00	0.00	0.00	0.00	0.00	0.00	0.00	0.00	0.01	9.57	0.00
**FinRW6**	0.00	0.00	0.00	0.00	0.00	0.00	0.00	0.00	21.65	0.00	0.00	59.22	0.00	0.00	0.00	0.01	5.02	0.00
**FinRW7**	0.00	0.06	0.02	0.00	0.00	0.00	0.00	0.00	0.08	0.00	0.00	0.00	0.00	0.00	0.01	6.12	0.21	0.01
**FinRW8**	0.00	0.00	0.00	0.00	0.00	0.00	0.00	0.00	0.00	0.00	0.00	0.00	0.00	0.00	0.01	0.01	21.34	0.00
**FinWWE5**	0.00	0.12	0.04	0.00	0.00	0.00	0.00	0.00	0.16	0.00	0.00	0.00	0.00	0.00	0.02	5.44	0.00	0.03
**FinWWE6**	0.00	0.00	0.04	0.00	0.00	0.00	0.00	0.00	0.00	0.01	0.00	0.00	0.00	0.00	0.02	4.00	7.02	0.03

**FinWWE7**	0.95	0.12	0.00	0.02	0.00	0.00	0.00	0.00	0.16	0.00	0.00	0.00	0.00	0.00	0.02	3.63	0.41	0.03
**FinWWE8**	0.00	0.12	2.50	0.00	0.00	0.00	0.00	0.00	0.16	0.00	0.00	0.00	0.00	0.00	0.02	7.82	0.41	0.03
**BcnSW2**	0.00	0.00	0.00	0.00	0.00	1.06	0.00	0.00	0.00	0.00	0.00	0.00	0.00	0.00	18.05	0.00	174.20	0.00
**BcnSW3**	0.00	0.00	0.00	0.00	0.00	0.00	0.00	0.00	0.00	0.00	0.00	0.00	0.00	0.00	0.05	0.04	141.08	0.00
**BcnRW3**	0.24	0.03	0.00	0.00	0.01	0.00	0.00	0.00	0.00	0.00	0.00	0.00	0.02	0.00	2.35	0.00	0.10	0.00
**BcnSW4**	0.00	0.00	0.00	0.00	0.00	0.00	0.00	0.00	0.00	0.00	0.00	0.28	0.00	0.00	10.14	0.00	115.71	0.00
**BcnSW5**	0.00	0.00	0.00	0.00	0.00	0.00	0.00	0.00	0.00	0.00	0.00	0.28	0.00	0.00	16.63	0.00	207.40	0.00
**BcnSW6**	0.00	0.00	0.00	0.00	0.00	0.00	0.00	0.00	0.00	0.00	0.00	0.28	0.00	0.00	13.27	0.00	126.68	0.00
**BcnSW7**	0.00	0.00	0.00	0.00	0.00	0.00	0.00	0.00	0.00	0.00	0.00	0.00	0.00	0.00	22.18	0.00	95.50	0.00
**BcnSW8**	0.00	0.00	0.00	0.04	0.00	0.00	0.00	0.00	0.00	0.00	0.00	0.00	0.01	0.00	15.42	0.00	121.51	0.00
**FrWWI1**	1.89	0.23	0.00	0.00	0.00	0.85	0.00	0.16	0.00	0.00	0.00	0.00	0.00	0.00	2.45	0.00	0.00	0.05
**FrWWI2**	53.68	0.23	0.00	0.03	0.00	0.85	0.00	0.16	0.00	0.00	0.00	0.00	0.00	0.00	1.49	0.00	0.00	0.05
**FrWWI3**	1.89	0.23	0.00	0.03	0.00	0.85	0.00	0.16	0.32	0.00	0.00	0.00	0.00	0.00	1.95	0.00	0.00	0.05
**FrWWI4**	1.89	0.23	0.00	0.00	0.00	0.00	0.00	0.00	0.00	0.00	0.00	0.00	0.00	0.00	2.34	0.00	0.00	0.05
**FrWWI5**	1.89	0.23	0.00	0.00	0.00	0.85	0.00	0.16	0.00	0.00	0.00	0.00	0.00	0.00	2.82	0.03	0.00	0.05
**FrWWI6**	1.89	0.23	0.00	0.00	0.00	0.85	0.00	0.16	0.00	0.00	0.00	0.00	0.00	0.00	1.77	0.00	0.00	0.05
**FrWWI7**	1.89	0.23	0.00	0.03	0.00	0.85	0.00	0.16	0.00	0.00	0.00	0.00	0.00	0.00	1.92	0.00	0.00	0.05
**FrWWI8**	0.00	0.23	0.00	0.00	0.00	0.85	0.00	0.16	0.00	0.00	0.00	0.00	0.00	0.00	1.43	0.00	0.00	0.05
**FrWWI9**	1.89	0.23	0.00	0.03	0.00	0.85	0.00	0.16	0.32	0.00	0.00	0.00	0.00	0.00	4.00	0.03	0.00	0.05
**ValWWI1**	1.89	0.23	0.00	0.00	0.00	0.85	0.00	0.16	0.00	0.00	0.00	93.63	0.00	0.00	2.78	0.00	0.82	0.00
**ValWWI2**	1.89	0.23	0.00	0.00	0.00	0.85	0.00	0.16	0.00	0.00	0.00	92.56	0.00	0.00	0.04	0.00	0.82	0.05
**ValWWI3**	62.98	0.23	0.00	0.00	0.00	0.85	0.00	0.16	0.00	0.00	0.00	92.57	0.00	0.00	0.04	0.03	0.82	0.05
**ValWWI4**	70.89	0.23	0.00	0.00	0.00	0.85	0.00	0.16	0.00	0.00	0.00	92.88	0.00	0.00	0.04	0.03	0.82	0.05
**ValWWI5**	1.89	0.23	0.00	0.00	0.00	0.85	0.00	0.16	0.00	0.00	0.00	93.35	0.00	0.00	0.04	0.03	0.82	0.05
**ValRW1**	0.00	0.00	0.00	0.00	0.00	0.00	0.00	0.00	0.08	0.00	0.00	47.08	0.00	0.00	0.01	0.00	0.21	0.00

ValRW2	0.00	0.00	0.00	0.00	0.00	0.00	0.00	0.00	0.08	0.00	0.00	47.19	0.00	0.00	0.01	0.00	0.21	0.00
ValRW3	0.00	0.06	0.00	0.00	0.00	0.00	0.00	0.00	0.08	0.00	0.00	47.45	0.00	0.00	0.01	0.00	0.21	0.00
ValRW4	0.00	0.00	0.00	0.00	0.00	0.00	0.00	0.00	0.08	0.00	0.00	48.06	0.00	0.00	0.01	0.00	0.21	0.00
ValRW5	0.00	0.06	0.00	0.00	0.00	0.00	0.00	0.04	0.08	0.00	0.00	46.81	0.00	0.00	0.01	0.00	0.21	0.00
FrWWE9	0.95	0.12	0.00	0.02	0.00	0.00	0.00	0.00	0.16	0.00	0.00	94.47	0.00	0.00	0.02	0.02	0.41	0.00
FrWWE10	53.29	0.12	0.00	0.02	0.00	0.42	0.00	0.08	0.00	0.00	0.00	94.71	0.00	0.00	2.54	0.02	0.00	0.03
FrWWE11	67.46	0.12	0.00	0.02	0.00	0.42	0.00	0.08	17.33	0.00	0.00	94.30	0.00	0.00	0.02	0.02	0.00	0.03
FrWWE12	55.54	0.12	0.00	0.02	0.00	0.00	0.00	0.08	0.16	0.00	0.00	0.00	0.00	0.00	0.02	0.00	0.41	0.03

Table S6. Detection and risk frequency of antibiotics analysed by target analysis in rivers, sea and samples collected near WWTPs.

Antibiotic	**Detection** [%]				**Risk** [%]*			
	**Sea water**	**River water**	**WWTP Influent**	**WWTP Effluent**	**Sea water**	**River water**	**WWTP Influent**	**WWTP Effluent**
Azithromycin	-	9.09	11.11	52.94	-	42.86	12.50	52.94
Clarithromycin	-	9.09	-	-	-	46.15	-	-
Erythromycin	-	3.03	-	-	-	-	-	-
Roxithromycin	-	9.09	-	-	-	-	-	-
Tilmicosin	-	9.09	-	-	-	83.33	-	-
Ciprofloxacin	-	7.58	-	-	-	45.45	-	-
Norfloxacin	-	6.06	-	-	-	100.00	-	-
Ofloxacin	-	3.03	-	-	-	100.00	-	-
Clindamycin	-	10.61	-	5.88	-	53.85	-	10.00
Lincomycin	-	1.52	-	-	-	100.00	-	-
Dimetridazole	-	7.58	-	-	-	-	-	-
Metronidazole	-	19.70	-	47.05	-	100.00	-	100.00
Hydroxymetronidazole	-	1.52	-	-	-	-	-	-
Sulfamethazine	-	6.06	-	-	-	-	-	-
Sulfamethoxazole	88.00	13.64	100.00	41.17	100.00	22.22	-	71.43
Sulfapyridine	-	18.18	-	-	-	-	-	-
Tetracycline	100.00	12.12	-	-	100.00	8.62	-	-
Trimethoprim	-	9.09	-	-	-	50.00	-	-

*This percentage is calculated by dividing the number of samples having an HQ >10 by the total number of samples having an HQ, i.e., greater than 0.1.

Table S7. Selected antibiotics and their internal standards parameters.

Antibiotic	Molecular formula	Molecular height [g/mol]	Antibiotic internal standard	Internal standard formula	Molecular weight [g/mol]
Azithromycin	$C_{38}H_{72}N_2O_{12}$	748.51	Azithromycin-d_3	$C_{38}H_{69}D_3N_2O_{12}$	751.53
Ciprofloxacin	$C_{17}H_{18}FN_3O_3$	331.13	Ciprofloxacin-d_8	$C_{17}H_{10}D_8FN_3O_3$	339.18
Clarithromycin	$C_{38}H_{69}NO_{13}$	747.48	Clarithromycin-d_3	$C_{38}H_{66}D_3NO_{13}$	750.50
Clindamycin	$C_{18}H_{33}ClN_2O_5S$	424.18	Clindamycin-^{13}C-d_3	$C_{18}{}^{13}CH_{30}D_3ClN_2O_5S$	444.20
Dimetridazole	$C_5H_7N_3O_2$	141.05	Dimetridazole-d_3	$C_5H_4D_3N_3O_2$	144.07
Erythromycin	$C_{37}H_{67}NO_{13}$	733.46	Erythromycin-d_3	$C_{37}H_{64}D_3NO_{13}$	736.48
Hydroxymetronidazole	$C_6H_9N_3O_4$	187.06	Hydroxymetronidazole-d_4	$C_6H_5D_4N_3O_4$	191.08
Lincomycin	$C_{18}H_{34}N_2O_6S$	406.21	Lincomycin-d_3	$C_{18}H_{31}D_3N_2O_6S$	409.23
Metronidazole	$C_6H_9N_3O_3$	171.06	Metronidazole-d_4	$C_6H_5D_4N_3O_3$	175.09
Norfloxacin	$C_{16}H_{18}FN_3O_3$	319.13	Norfloxacin-d_8	$C_{16}H_{10}D_8FN_3O_3$	327.18
Ofloxacin	$C_{18}H_{20}FN_3O_4$	361.14	Ofloxacin-d_8	$C_{18}H_{12}D_8FN_3O_4$	369.19
Roxithromycin	$C_{41}H_{76}N_2O_{15}$	836.52	Roxithromycin-d_7	$C_{41}H_{69}D_7N_2O_{15}$	843.57
Sulfamethazine	$C_{12}H_{14}N_4O_2S$	278.08	Sulfamethazine-d_4	$C_{12}H_{10}D_4N_4O_2S$	282.11
Sulfamethoxazole	$C_{10}H_{11}N_3O_3S$	253.05	Sulfamethoxazole-d_4	$C_{10}H_7D_4N_3O_3S$	257.08
Sulfapyridine	$C_{11}H_{11}N_3O_2S$	249.06	Sulfapyridine-d_4	$C_{11}H_7D_4N_3O_2S$	253.08
Tetracycline	$C_{22}H_{24}N_2O_8$	444.15	Tetracyclin-d_6	$C_{22}H_{18}D_6N_2O_8$	450.19
Tilmicosin	$C_{46}H_{80}N_2O_{13}$	868.57	Tilmicosin-d_3	$C_{46}H_{77}D_3N_2O_{13}$	871.58
Trimethoprim	$C_{14}H_{18}N_4O_3$	290.14	Trimethoprim-d_3	$C_{38}H_{69}D_3N_2O_{12}$	293.16
